# Genetic and health determinants of cancer risk in Bangladeshi and Pakistani individuals in the UK

**DOI:** 10.1038/s41467-026-74415-z

**Published:** 2026-06-18

**Authors:** Abu Zafer Mohammed Dayem Ullah, Ashitha Joby, Graeme J. Thorn, Lewis G. E. James, Isabelle Sanders, David A. van Heel, Genes & Health Research Team, Shabana Chaudhary, Joseph Gafton, Karen A. Hunt, Shapna Hussain, Kamrul Islam, Mohammed Bodrul Mazid, Elizabeth Owor, Jessry Russell, Nishat Safa, John Solly, Marie Spreckley, David A. van Heel, Jan Whalley, Ishevanhu Zengeya, Emily Mantle, Eamonn Maher, Ana Angel, Saeed Bidi, Fabiola Eto, Sarah Finer, Chris Griffiths, Sam Hodgson, Benjamin M. Jacobs, Rohini Mathur, Caroline Morton, Asma Qureshi, Stuart Rison, Annum Salman, Miriam Samuel, Moneeza K. Siddiqui, Daniel Stow, Sabina Yasmin, Julia Zöllner, Sheik Dowlut, Shaheen Akhtar, Samina Ashraf, Dan Mason, John Wright, Michael Simpson, Richard C. Trembath, Gerome Breen, Raymond Chung, Sang Hyuck Lee, Daniel MacArthur, Omar Asgar, Joanne Harvey, Karen Tricker, Caroline Winckley, Hanifa Khatun, Amna Asif, Claudia Langenberg, Grainne Colligan, Ceri Durham, Bill Newman, Ahsan Khan, Hilary Martin, Teng Heng, Matt Hurles, Vivek Iyer, Georgios Kalantzis, Vladimir Ovchinnikov, Iaroslav Popov, Klaudia Walter, Panos Deloukas, David Collier, Claude Chelala

**Affiliations:** 1 Centre for Cancer Biomarkers and Biotherapeutics, Barts Cancer Institute, Queen Mary University of London, London, UK; 2 Blizard Institute, Queen Mary University of London, London, UK; 3 Aston University, Birmingham, UK; 4 Wolfson Institute of Population Health, Queen Mary University of London, London, UK; 5 Bradford Teaching Hospitals NHS Foundation Trust, Bradford, UK; 6 King’s College London, London, UK; 7 Garvan Institute of Medical Research, Darlinghurst, NSW Australia; 8 Manchester University Hospitals, Manchester, UK; 9 Precision Healthcare University Research Institute, Queen Mary University of London, London, UK; 10 Social Action for Health, London, UK; 11 University of Manchester, Manchester, UK; 12 Waltham Forest Council, London, UK; 13 Wellcome Sanger Institute, London, UK; 14 William Harvey Research Institute, Queen Mary University of London, London, UK

**Keywords:** Computational biology and bioinformatics, Cancer genomics, Genetics research, Risk factors

## Abstract

South Asian populations remain underrepresented in cancer genomics, despite elevated risk for certain malignancies and distinct clinical profiles. This gap is especially pronounced for British Bangladeshi and Pakistani communities. We analyse data from 57,416 individuals of Bangladeshi and Pakistani ancestry in the UK-based *Genes & Health* cohort, integrating electronic health records, cancer registry data, and whole-exome sequencing (n = 43,462). Among them, 2,782 (4.8%) has cancer, with earlier onset across multiple types compared to national benchmarks. A phenome-wide case–control analysis identifies 132 significant cancer–comorbidity associations, with larger effect sizes observed for topographically concordant cancer-comorbidity pairs, and stronger associations for systemic disorders such as obesity and hypertension. Exome-wide analyses reveal 39 variant-level and 31 gene-level associations, over 60% absent from major genomic databases assessed and enriched for ultra-rare coding variations. Notable loci included *ARHGAP45*, *CBR1*, *FKBP6*, *NF1*, and *ZNF155*, with several ancestry-specific associations. These findings highlight distinct cancer risk architectures in this South Asian subpopulation, emphasising the need for population-tailored risk models and screening strategies.

## Abstract (web summary)

South Asian populations remain underrepresented in cancer genomics, thus increasing health inequities. Here, the authors analyse data from 57,416 individuals of Bangladeshi and Pakistani ancestry in the UK-based Genes & Health cohort, finding ancestry-associated cancer risk variants and cancer-comorbidity pairs.

## Introduction

Cancer continues to be one of the leading global health challenges, with an estimated 20 million new cases and 10 million deaths in 2022 alone^1^. Despite considerable advances in prevention, early detection, and treatment, substantial disparities persist in cancer incidence, presentation and survival outcomes across population groups identified by geographical region, ethnicity or income level^1,2^. People of South Asian ancestry, who comprise nearly a quarter of the global population and are one of the fastest-growing minority groups in Western countries—including the United Kingdom (8.5%), Canada (7.1%), Australia (6.6%), and the United States (1.5%)—remain severely underrepresented in healthcare research and genomic studies^3–6^.

Historically, South Asian migrants to Western nations have exhibited lower overall cancer incidence compared to their host populations, particularly for malignancies such as breast, lung, colorectal, and prostate cancer—diseases that collectively account for a substantial proportion of cancer morbidity and mortality in Western countries^7–9^. However, growing evidence indicates a rapid convergence in cancer incidence rates between South Asians and their host populations, especially with increasing age and length of residence^10^. Furthermore, South Asian patients are disproportionately diagnosed at younger ages or with more advanced-stage disease compared to their White counterparts^9,11,12^, a disparity often attributed to lower participation in routine cancer screening programmes^13–15^. While socio-cultural barriers and health care system factors contribute to these disparities, such reasoning may not fully account for the observed differences in cancers that lack standardised population-based screening protocols.

South Asians experience a disproportionately high burden of chronic health conditions, including type 2 diabetes mellitus, metabolic syndrome, central obesity, and chronic viral hepatitis^16–22^. These conditions are well-established risk factors for several malignancies—notably liver, colorectal, pancreatic, and endometrial cancers—and are thought to have a strong inherited genetic component in South Asian populations^23,24^. However, our understanding of the genetic basis of complex diseases, including cancer, remains largely biased towards populations of European ancestry^25^. To date, individuals of South Asian descent have constituted only about 2% of participants in published genome-wide association studies (GWAS)^24^. Large, diverse resources such as the UK Biobank (~1%), the All of Us Research Programme (~1%), and the Mass General Brigham Biobank (<1%) still include insufficient South Asian representation, limiting the ability to conduct meaningful genetic analyses of cancer risk^26^. This persistent underrepresentation significantly limits our ability to fully understand cancer biology, improve risk prediction, and develop personalised therapeutic strategies for this growing, diverse population.

Understanding the intersection between clinical risk factors and genetic predisposition in South Asian populations can uncover valuable insights for cancer prevention and management tailored to this group. Such understanding is crucial for developing culturally relevant prevention strategies, enhancing early detection, and creating risk models specific to this population. To advance this objective, the present study uses the *Genes & Health* (G&H) cohort—the largest longitudinal genotype–phenotype resource of Bangladeshi and Pakistani individuals recruited in the United Kingdom. This cohort includes ~57,000 participants^27^, with around 2800 cancer cases across 33 cancer types encompassing both primary sites and broader organ systems. We also assess whether our ancestry-specific findings are consistent with patterns observed in the cancer cohort of Genomics England (GEL) 100,000 Genomes Project (100K GP)^28^.

Here, we conduct a comprehensive characterisation of cancer phenotypes and associated clinical risk factors within this cohort, leveraging extensive electronic health records and cancer registry data. We explore the contribution of rare protein-coding genetic variants in cancer susceptibility using whole-exome-sequencing data from approximately 43,000 individuals, and evaluate these genetic associations in the context of existing GWAS studies, biomarker and drug target databases. We identify 132 significant cancer-comorbidity associations and 39 rare variant associations across multiple cancer types, with over 60% absent from European-ancestry studies. These results fill critical gaps in the current literature by elucidating the combined effects of clinical and inherited risk factors for cancer in an underrepresented ethnic group, providing a foundation for population-specific biomarker discovery and informing strategies for risk stratification, screening protocols, and personalised therapeutic approaches for South Asian communities in the Western world.

## Results

### Cancer incidence and age at diagnosis

**Fig. 1:**
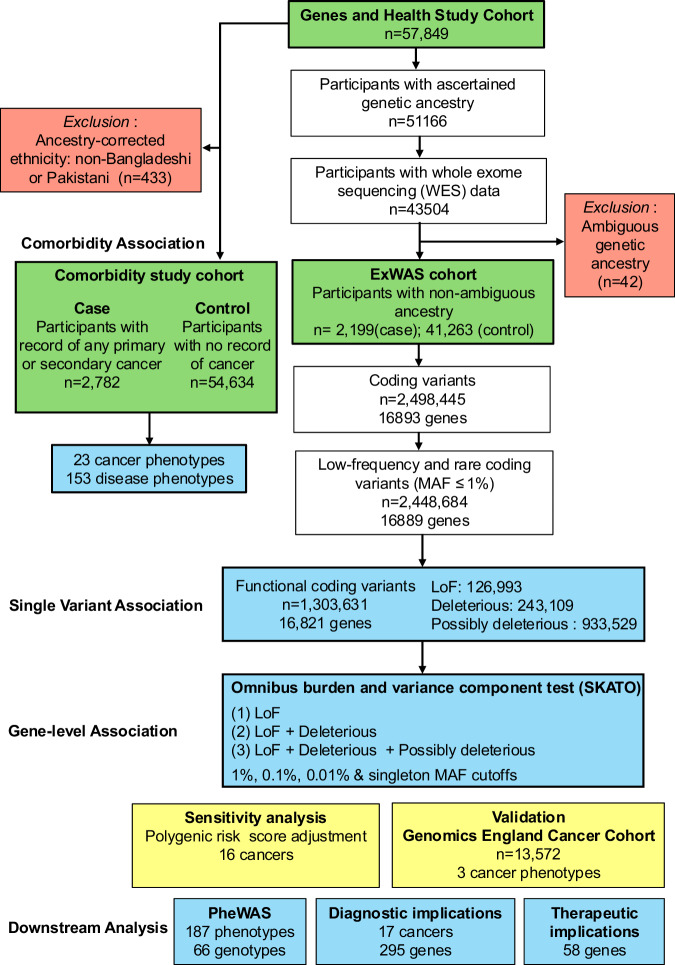
Study overview for germline variants and clinical determinants exploration across cancer phenotypes in British Bangladeshi and Pakistani individuals. Two streams of analysis were conducted on the Genes & Health study cohort (*n* = 57,416). Comorbidity association stream focused on exploring associations between cancer phenotypes and pre-existing non-cancer medical conditions. Exome-wide association study (ExWAS) stream focused on discovering associations between cancers and rare loss-of-function and missense coding variants, at the single-variant as well as gene level, using a subset of participants with whole-exome-sequenced data (*n* = 43,462). Significantly associated genes and/or variants were further tested for differential germline profile in Genomics England Cancer Cohort (*n* = 13,572), association with other phenotypes, and viability in germline testing and known treatment regimens.

The G&H cohort included 57,416 South Asian individuals of Bangladeshi and Pakistani origin (SAS-BP), of whom 2782 (4.85%) had a cancer diagnosis recorded in their electronic health records (EHR) (Fig. 1). A high degree of concordance (99%) was observed between EHR-coded ethnicity and genetic Ancestry (gAncestry) derived from genotyping array data for 51,166 participants (Supplementary Fig. 1), which aligns with the recruitment criteria of this cohort. After correcting for gAncestry information, i.e., using unambiguous gAncestry information where available as confirmed ethnic origin, and reported ethnicity for 6312 participants (11%) with ambiguous or missing gAncestry information, the study cohort comprised 52% Bangladeshi and 48% Pakistani origin participants (Supplementary Data 1).

**Fig. 2:**
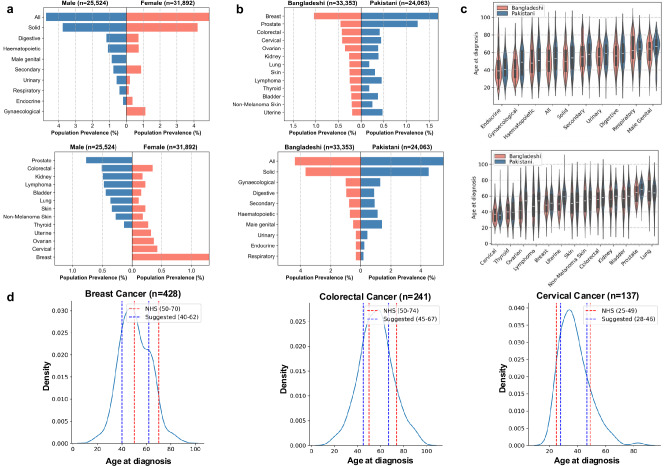
Demographic features of cancer patients within the Genes & Health study cohort. **a** and **b** Bi-directional barplots showing prevalence of different cancers within the study cohort, stratified by gender and ethnicity. Each bar represents the prevalence of a cancer in the specific demographic subgroup—male, female, Bangladeshi, Pakistani. Reported ethnicity of participants is corrected for genetic ancestry determined from genotyping array data, where available. Barplots are displayed in descending order of prevalence in male and Bangladeshi subgroups (from top to bottom). **c** Grouped violin plots with overlaid boxplots showing the distribution of age of cancer diagnosis, stratified by genetic ancestry corrected ethnicity. All boxplots show median (centre), 25th percentile (bottom of box), 75th percentile (top of box), smallest/largest value within 1.5 × interquartile range from hinge (bottom/top whiskers, respectively). Plots are displayed in ascending order of median diagnosis age for cancers in the Bangladeshi subgroup (from left to right). **a–c** Each pair of plots shows demographic distribution for cancers of 13 primary sites (bottom), and 7 organ systems and 3 aggregate types (top). The number of participants in each cancer are: 2782 (all), 2312 (solid), 534 (digestive), 515 (haematopoietic), 479 (secondary), 428 (breast), 361 (gynaecological), 241 (colorectal), 222 (male genital), 212 (urinary), 196 (prostate), 193 (lymphoma), 182 (kidney), 164 (endocrine), 163 (bladder), 159 (skin), 149 (respiratory), 137 (cervical), 130 (lung), 129 (non-melanoma skin), 121 (thyroid), 117 (ovarian) and 102 (uterine). **d** Visualisation of the age-based screening windows for breast, colorectal and cervical cancers in the British Bangladeshi and Pakistani populations. The red vertical lines show current screening windows deployed in the UK National Health Service. The blue vertical lines show the suggested windows as dictated by this study.

Cancers originating from the gynaecological, digestive, and hematopoietic systems were more prevalent, with breast, prostate, cervical and colorectal cancers representing the most prevalent primary sites (Supplementary Data 2). The prevalence of 13 distinct primary, 7 organ-system and 3 aggregate cancer types within the cohort stratified by demographic characteristics is shown in Fig. 2. Excluding gender-specific cancers, the overall incidence of cancer was generally higher in men than women. However, the prevalence of (female) breast cancer was notably high in this population (1.3%), which skewed the overall cancer statistics towards women.

When comparing Bangladeshi and Pakistani participants, the latter exhibited a higher cancer diagnosis rate, with the disparity being particularly pronounced for breast, reproductive organs and lymphoma cancers. However, Bangladeshi participants were diagnosed at a younger median age for these cancer types (Breast: 48 years vs. 51 years; Ovarian: 42 years vs. 54 years; Uterine: 50 years vs. 59 years; Prostate: 64.5 years vs. 68.5 years; Lymphoma: 46 years vs. 54 years). Cervical cancer patients were the youngest group, with a median age of 36 years (interquartile range, IQR = 13 years), while prostate cancer patients were the oldest, with a median age of 67 years (IQR = 14 years).

Approximately half of the cancer patients in our cohort were diagnosed by the age of 51 (IQR:24 years) (Fig. 2c), which is notably earlier than the broader, predominantly White population in the country. In the general UK population, only 14% of cancer patients are diagnosed by the age of 50^29^. This early onset was also evident in the GEL cohort (Supplementary Data 3), where the median age of cancer diagnosis was 64 years (IQR = 18 years) for Europeans and 57 years (IQR = 19 years) for South Asians outside of Bangladeshi or Pakistani ancestry (SAS-nBP; presumably largely Indian ancestry). This earlier age of diagnosis was particularly pronounced for breast, ovarian, uterine, colorectal, and respiratory cancers. To assess whether the current UK NHS screening guidelines are equitable for this cohort^30^, we further stratified the breast, colorectal, and cervical cancer groups based on the central 60% of the age distribution (Fig. 2d). We identified cancer-specific intervals: 40–62 years for breast cancer, 45–67 years for colorectal cancer, and 28–46 years for cervical cancer. These findings suggest that bringing forward the screening windows for breast and colorectal cancers by approximately 10 and 5 years, respectively, would provide equitable benefit for the SAS-BP population. Interestingly, a narrower screening window for cervical cancer would yield similar benefits.

### Comorbidities associated with cancer

To explore pre-existing non-malignant conditions that might contribute to or reflect cancer susceptibility, we conducted a case-control study to examine associations between 23 cancer types and 151 phenotypes. Comorbidities were defined using a validated phecode framework, which mapped diagnostic codes from both primary care and secondary care into clinically meaningful categories (see the “Methods” section).

The G&H cohort is characterised by a high rate of kinship, with 36.5% of participants being first cousins or closer^31^. The rate was even higher among the cancer cases, with 61.1% having at least one second-degree or closer relative (Supplementary Data 1). Given this familial structure, associations were estimated using a mixed-model framework implemented in SAIGE with a sparse genetic relationship matrix derived from KING kinship estimates. This relatedness-aware modelling approach was used throughout the analysis to ensure that association estimates were robust to familial clustering within the cohort.

**Fig. 3:**
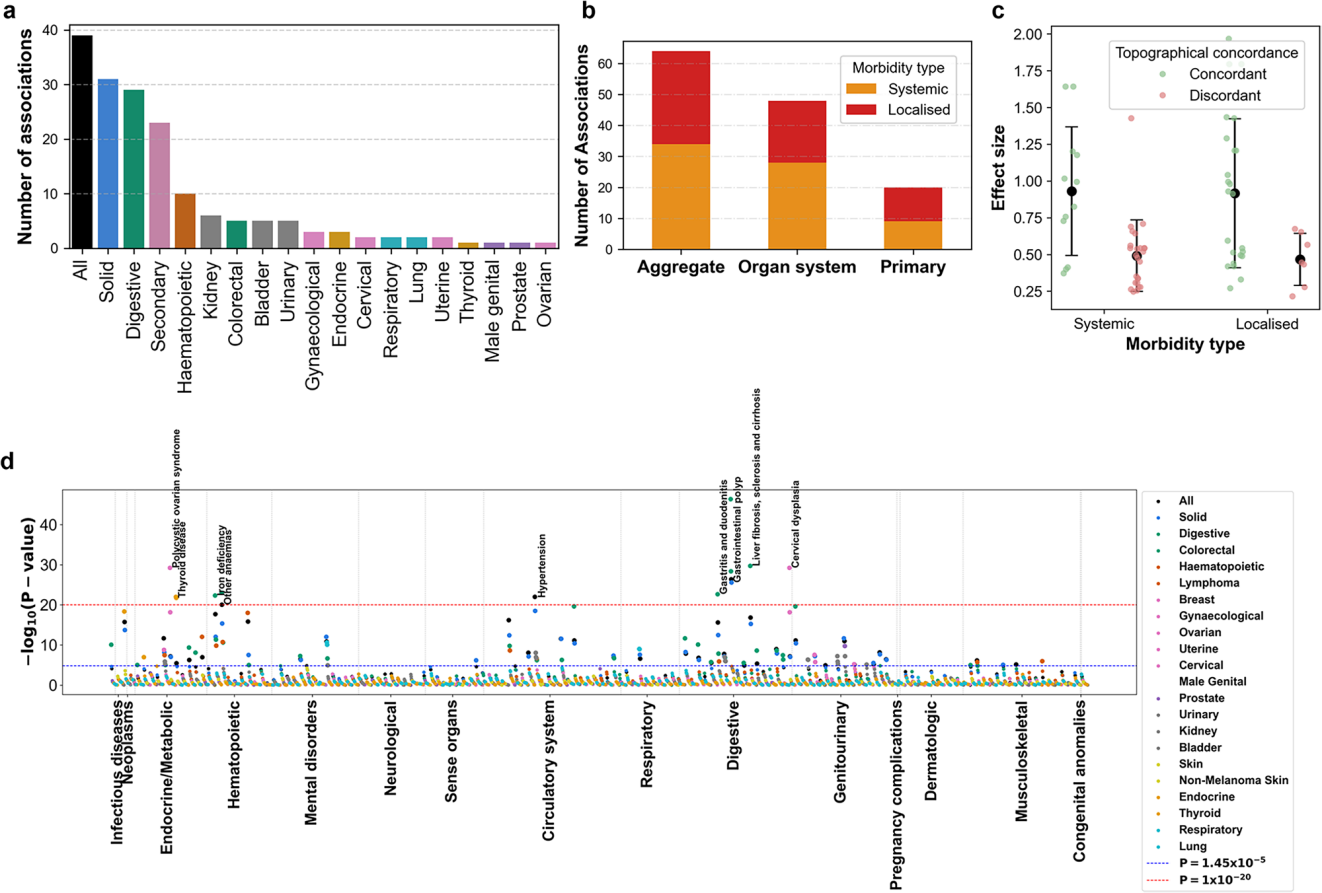
Summary of comorbidity association study results. **a** Barplot showing number of significant associations found between each cancer type and pre-existing medical conditions. **b** Distribution of systemic and localised pre-existing conditions significantly associated with cancers, grouped by topographical distribution (primary—cancers in a single organ; organ system—cancers in multiple primary sites from the same organ system; Aggregate—*all*, *solid* and *secondary* cancers with heterogeneous origins). **c** Strip plot with mean ± standard deviation showing effect size of associations, grouped by types of medical conditions: systemic (*n* = 37 associations) vs. localised (*n* = 31 associations) and topographical alignment: concordant (*n* = 36 associations) vs. discordant (*n* = 32 associations). The *y*-axis shows the log-scaled odds ratio, with each dot representing a cancer-comorbidity association. Associations with aggregate cancers were excluded. **d** Manhattan plot showing associations between 23 cancers and 147 pre-existing medical conditions stratified by 14 phenotype groups. Associations were tested using the SAIGE score test with saddlepoint approximation; all *P* values are two-sided. The blue dashed line represents the Bonferroni-corrected significance threshold at *P*  =  1.45 × 10^−5^. Points (associations) are colour-coded according to cancer type. Only pre-existing conditions from highly significant associations (above red line; *P*  ≤ 1 × 10^−20^) are labelled (*n* = 15 associations).

A total of 3442 tests were performed. After applying Bonferroni correction for multiple testing with a significance threshold of *P* ≤ 1.45 × 10^−5^, we identified 146 significant associations for aggregate cancer types and 25 for primary cancer types. Most of these significant associations were observed in the top five aggregate cancer types (Fig. 3a). Several cancer pairs exhibited a high degree of correlation (Pearson’s *r* > 0.8), including all—solid, endocrine—thyroid, respiratory—lung, prostate—male genital, skin—non-melanoma skin, bladder—urinary, and kidney—urinary cancers. In cases where a common signal emerged from these pairs, we retained the association with the lower *P*-value, resulting in 132 unique cancer–phenotype associations (Supplementary Data 4).

The top five significant associations identified were gastrointestinal polyp, chronic liver disease or anaemia prior to digestive cancer (*P* = 4.3 × 10^−47^, *P* = 1.9 × 10^−30^, *P* = 9.4 × 10^−24^), and cervical dysplasia or polycystic ovarian syndrome prior to cervical cancer (*P* = 5.8 × 10^−30^, *P* = 5.8 × 10^−30^). The largest effect size was observed for benign tumour in the central nervous system (CNS) prior to endocrine cancer (odds ratio, OR = 7.1 [95% confidence interval, CI = 4.6–11.0], *P* = 4.5 × 10^−19^). Among systemic disorders, obesity, hypertension, cardiovascular diseases, chronic renal disease and anaemias accounted for 49% (35 of 71) of the associations. Notably, hypertension and obesity, two highly prevalent conditions in the community (26% and 21%, respectively), were associated with increased odds of digestive, haematopoietic and renal cancers.

Across the cancer spectrum, the highest number of pre-existing conditions was observed for digestive system diseases/disorders (24%), followed by genitourinary system conditions (17%) and circulatory system diseases (16%) (Fig. 3d). After excluding associations related to three aggregate cancers encompassing heterogeneous sites, 36 of the remaining 68 associations (53%) involved pre-existing conditions affecting the same anatomical location or organ system as the corresponding cancer (topographical concordance) and generally showed stronger effect sizes. This pattern suggests the impact of exposure to localised disorders on carcinogenesis within the same organ system (Fig. 3b and c). In contrast, systemic conditions occurring in anatomically distinct systems (topographically discordant conditions) tended to show weaker associations across cancer types. In general, effect sizes among significant associations revealed an inverse prevalence-effect relationship: conditions with <1% prevalence showed median OR = 2.4 (range = 1.4–7.1), compared to median OR = 1.4 (range = 1.1–1.9) for conditions with 10–20% prevalence.

Most associations with systemic disorders align with known risk factors for the corresponding cancers. One notable finding was the association between fibromyalgia with haematopoietic (OR = 1.9 [1.5–2.5], *P* = 1.9 × 10^−6^) and digestive cancers (OR = 1.7 [1.4–2.2], *P* = 1.4 × 10^−5^). While fibromyalgia, a chronic pain disorder, shares overlapping symptoms with cancer, such as pain and fatigue, no direct link has been established. Our findings may reflect a misdiagnosis of cancer as fibromyalgia, a common issue due to the crossover in symptoms^32^. We also observed a strong association between substance misuse and respiratory cancer (OR = 2.0 [1.6–2.4], *P* = 2.3 × 10^−11^), as well as with digestive and bladder cancers.

Subgroup analysis by gender and ethnicity identified 24 significant associations specific to either male or Bangladeshi participants (Supplementary Data 5). These results suggest an opportunity to expand surveillance efforts for these groups. Specific associations of interest included: Bangladeshi participants—gastritis/duodenitis in digestive cancer (OR = 1.8 [1.6–2.1], *P* = 1.3 × 10^−21^), peripheral arterial disease in bladder cancer (OR = 2.9 [1.9–4.6], *P* = 3.4 × 10^−6^), obesity in urinary cancer (OR = 1.8 [1.4–2.4], *P* = 2.9 × 10^−6^); SAS-BP men—iron deficiency in colorectal cancer (OR = 2.3 [1.8–2.9], *P* = 4.9 × 10^−12^), COPD in respiratory cancer (OR = 2.2 [1.7–2.8)], *P* = 7.5 × 10^−10^) and hypertension in digestive cancer (OR = 1.4 [1.2–1.6], *P* = 5.1 × 10^−7^).

### Coding variant distributions

We analysed whole-exome sequencing (WES) data from 43,462 individuals within G&H, following rigorous quality control procedures and excluding participants with ambiguous gAncestry (see the “Methods” section). The cohort included 2199 cancer patients (5.1%) and 41,263 cancer-free participants. 54% of the participants were of Bangladeshi gAncestry, which closely mirrors the composition of the overall study population. We identified 2.5 million autosomal variants within the coding regions of genes (Table 1), of which 98% were classified as rare (variants up to minor allele frequency (MAF) = 1% across the cohort; Supplementary Data 6). This catalogue represents a 4.2-fold increase over the number of South Asian participants in the UK Biobank dataset, with a 1.4-fold increase in the number of coding variations^33^.

**Table 1:** Coding variants discovered in exome-sequencing data from the Genes & Health WES cohort

	Genes & Health (*n* = 43,462)	Variants not observed in gnomAD 4.1 Exomes		
Variant category	Number of variants, *N* (% with MAC = 1)	Variants per participant, median (IQR)	South Asian (*n* = 43,129) *N* (%)	All (*n* = 730,947) *N* (%)
Coding regions	2,498,445 (42.76)	18,373 (305)	1,155,378 (46.24)	695,288 (27.83)
**Predicted function**				
LoF	127,662 (53.76)	191 (15)	81,570 (63.90)	61,940 (48.52)
Start lost	3033 (49.65)	7 (2)	1743 (57.47)	995 (32.81)
Stop gained	43,145 (52.23)	52 (8)	25,054 (58.07)	15,808 (36.64)
Stop lost	1319 (51.32)	13 (3)	777 (58.91)	606 (45.94)
Splice donor	14,203 (53.47)	13 (4)	9032 (63.59)	6404 (45.09
Frameshift	55,489 (54.97)	91 (10)	37,993 (68.47)	32,644 (58.83)
Splice acceptor	10,473 (55.56)	14 (3)	6971 (66.56)	5483 (52.35)
Missense	1,553,669 (43.73)	8354 (163)	734,794 (47.29)	441,008 (28.38)
Deleterious	243,109 (48.08)	79 (11)	125,796 (51.74)	74,776 (30.76)
Possibly deleterious	933,529 (44.23)	2138 (66)	447,120 (47.90)	271,557 (29.09)
Likely benign	377,031 (39.70)	6132 (117)	161,878 (42.93)	94,675 (25.11)
In-frame indels	31,711 (43.26)	199 (13)	17,082 (53.87)	12,411 (39.14)
Insertion	8742 (41.85)	91(9)	4997 (57.16)	4108 (46.99)
Deletion	22,969 (43.79)	108 (10)	12,085 (52.61)	83,030 (36.15)
Synonymous	785,369 (39.02)	9621 (161)	321,915 (40.99)	179,914 (22.91)
Other coding	34 (38.23)	1 (0)	17 (50.00)	15 (44.12)

Variant functions are predicted based on mapping to the canonical transcript

*MAC* minor allele count, *IQR* interquartile range (quartile 3−quartile 1)

Among the variants identified, 1,553,669 were missense (median of 8354 per individual), and 127,662 were putative loss-of-function (LoF) variants (median of 191 per individual) (Table 1). Of the missense variants, ~16% (243,109) were predicted to be deleterious by 5 prediction algorithms (henceforth referred to as ‘deleterious missense’ variants) while 60% (933,529) were predicted to be deleterious by at least one algorithm (henceforth referred to as ‘possibly deleterious missense’ variants). Notably, ~43% of variants were observed only once in the dataset (singleton variants; Supplementary Fig. 4).

While variant distributions showed little to no difference between cancer and non-cancer groups across variant types, significant differences were observed between Bangladeshi and Pakistani participants, with a higher number of variants identified in the Bangladeshi group (median: 18,451 vs. 18,204; Supplementary Data 7). Almost half of these variants were not reported in gnomAD v4.1 South Asian Exomes, and a quarter were not reported in any ancestry group (Table 1). This pattern was even more pronounced for LoF variants, with 64% unseen in South Asian populations and 49% overall. This distinctive catalogue of coding variants, coupled with the relatively large sample size, provides a unique opportunity to investigate germline genetics in cancer within this under-researched ethnic group.

### Coding variant associations

We conducted a variant-level exome-wide association study (ExWAS) to investigate associations between 22 cancer phenotypes (excluding secondary cancers) and rare coding variants observed in at least five participants, resulting in 6.2 million individual tests. The smallest cancer cohort, uterine cancer, included 76 cases. The analysis focused on 331,291 LoF variants (including stop gain, stop lost, start lost, frameshift and essential splice variants), as well as deleterious and possibly deleterious missense variants across 16,422 protein-coding genes.

**Fig. 4:**
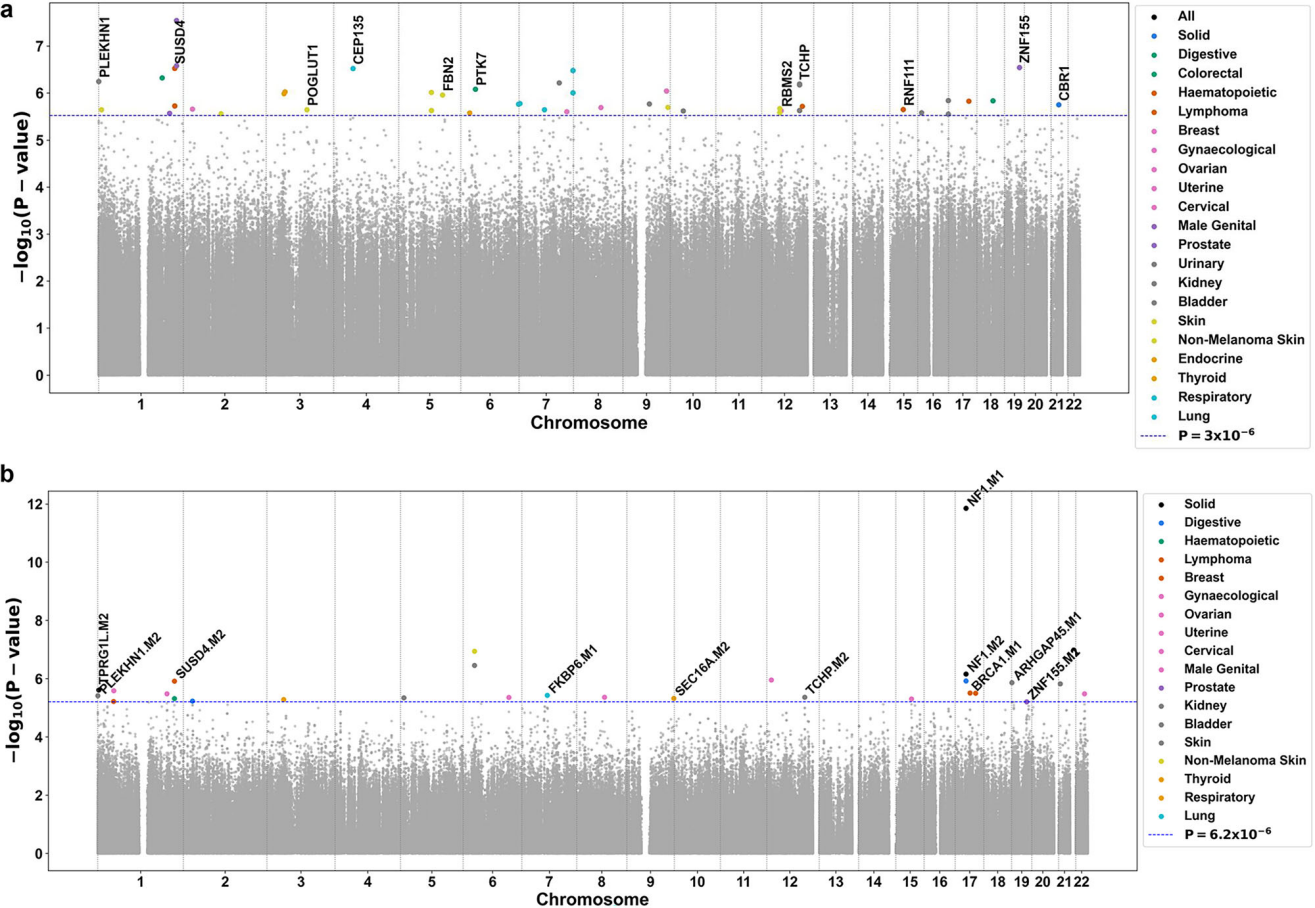
Summary of exome-wide association study results. Multi-trait Manhattan plot exhibiting the landscape of **a** variant–cancer and **b** gene–cancer associations across the 331,291 LoF, deleterious and possibly deleterious missense coding variants, with the −log_10_ of the *P-*value on the *y-*axis. Associations were tested using REGENIE: **a** single-variant association using logistic regression under an additive genetic model, and **b** gene-level associations using SKAT-O burden test; all *P* values are two-sided. The blue dashed line represents the study-specific significance threshold at **a** *P* = 3 × 10^−6^ and **b** *P* = 6.2 × 10^−6^ to account for multiple hypothesis testing. Each point (association) above the significance threshold is colour-coded according to cancer phenotype. **a** Only genes representing LoF or deleterious missense variants are labelled. **b** A maximum of 3 masks per gene was tested, representing aggregation of (M1) LoF, (M2) LoF + deleterious missense, (M3) LoF + deleterious + likely deleterious missense variants at decreasing MAF cutoffs of 1%, 0.1%, 0.01% and singletons. For significant gene–cancer associations that appear in multiple MAF thresholds, the association with the highest significance was plotted. Only gene–cancer associations at M1 and M2 masks are labelled.

Given the high degree of relatedness within the cohort, association analyses were performed using the whole-genome regression approach implemented in REGENIE, which accounts for relatedness, population structure and polygenicity. This approach also employs a fast, approximate Firth regression approach for binary outcomes. Using a *P*-value threshold of 3 × 10^−6^ (see the “Methods” section), we identified 47 significant associations (Fig. 4a; Supplementary Data 8). In cases where a common signal was observed from highly correlated cancer pairs, we retained the association with the lower *P*-value, resulting in 39 unique cancer-variant associations. Of these, 38 were derived from ultra-rare variants (MAF ≤ 0.1%). 28% (11 of 39) of these variants were absent in gnomAD South Asian exomes. The rarest significant variant was a deleterious missense variant in the Carbonyl Reductase 1 gene (*CBR1*:*p*.Arg74Pro), which plays a critical role in drug metabolism and was associated with aggregated solid cancers (cohort MAF of 0.006%). All significant associations exhibited a consistent positive direction of effect, contrasting with disease-associated non-coding variants, which can variably affect gene expression^34^. The largest effect was observed for a frameshift variant in the Zinc Finger Protein 155 gene (*ZNF155*), which was associated with prostate cancer (log-OR = 7.0 [5.0–9.1], *P* = 2.8 × 10^−7^)—the only significant LoF variant association. Analogous to comorbidity analyses, a strong inverse correlation between MAF and effect magnitude was observed (Spearman *ρ* = −0.72, *P* < 10⁻⁸), with ultra-rare variants showing larger effects (median log-OR = 5.1) compared to rare variants (MAF 0.1–1%; median log-OR = 3.3).

Genes representing 62% (24 of 39) of the associations were not annotated with cancer associations in the OMIM, GWAS catalogue, AstraZeneca PheWAS (AZ-PheWAS), or FinnGen release 12 databases. In total, 21 gene–cancer relationships were identified across these databases: GWAS catalogue (*n* = 10), AZ-PheWAS (*n* = 7), and FinnGen (*n* = 4) databases. However, all 10 GWAS catalogue associations represented non-coding or synonymous variants; In contrast, 9 missense or LoF variant associations were identified from AZ-PheWAS and FinnGen by applying a more lenient significance threshold of *P* ≤ 1 × 10^−5^. Of these, two (*ANKRD11*, *KRT81*) passed with a suggestive threshold of *P* ≤ 1 × 10^−6^.

The subgroup analysis revealed that the majority of cohort-wide significant LoF and deleterious variant associations were driven by either of the ancestry subgroups (Supplementary Data 9). However, two associations were observed specifically in the Pakistani subgroup and did not reach significance at the cohort level: a splice-site variant in the *TRPM2* gene associated with lymphoma and a deleterious missense variant in *METTL13* (Ser425Pro), a known oncogene, associated with solid cancer. Notably, *CBR1*:*p*.Arg74Pro was the only cohort-wide significant variant whose association with solid cancer was not replicated in any of the ancestry subgroups. This lack of replication was possibly due to the ultra-rare nature of the variant, which did not pass the stringent minor allele count filter of five in any of the subgroups.

### Gene-level variant aggregation and associations

We performed gene-level association analyses by aggregating the effect of qualifying protein-altering variants, employing the omnibus strategy SKAT-O that combines the traditional gene burden test with the variance component test (see the “Methods” section). We applied 12 distinct sets of qualifying variant filters (masks) to test the association of 22 cancer phenotypes with 16,821 genes. The 12 masks represented the aggregation of (i) LoF, (ii) LoF + deleterious missense, (iii) LoF + deleterious and possibly deleterious missense variants at MAF cutoffs of 1%, 0.1%, 0.01% and singletons.

Using a significance threshold of *P* ≤ 6.2 × 10^−6^ (see the “Methods” section), we identified 31 significant associations, involving 25 genes and 28 gene–cancer pairs. 94% of these associations were driven by the accumulation of ultra-rare variants, including singletons (Fig. 4b; Supplementary Data 10). The most significant association was observed for singleton LoF variants in *NF1*, which encodes the tumour suppressor protein neurofibromin, and was associated with adult solid cancers (*P* = 1.4 × 10^−12^). Other genes where LoF variants were associated with increased cancer risk included *ARHGAP45* (skin cancer); *BRCA1* (breast cancer); *FKBP6* (lung cancer); and *ZNF155* (prostate cancer). In all cases, the directions of effect were positive, meaning the aggregation of variants increases cancer risk, with the exception of *ARID2*, a tumour suppressor gene, which was associated with a decreased risk of all cancer combined (*P* = 1.9 × 10^−6^). LoF-only gene masks yielded larger effects (median log-OR = 4.7) compared to masks incorporating deleterious missense variants (median log-OR = 2.2).

As with the variant-level analysis, 64% (16 of 25) of the significant genes lacked documented associations with cancer in the public databases assessed. Only two associations were directly supported by existing databases: the *BRCA1* gene with breast cancer (OMIM and AZ-PheWAS) and the *NF1* gene with overall cancer (AZ-PheWAS). For eight genes (*HLA-DMB, LAMB2, NF1, PKP1, SEC16A, SKA2, TMPRSS15, ZBTB10*), our findings extended documented cancer associations by implicating these loci in additional cancer types beyond those previously described. We also identified cancer associations for a further 16 genes without corresponding cancer annotations in the databases assessed. The large proportion of associations not represented in current genomic resources underscores the value of our findings and provides insights into the phenotypic consequences of protein-altering variants in the South Asian population, potentially revealing additional therapeutic targets.

Among the significant gene-level associations, only 25% (7 of 28) were also detectable via variant-level analysis. This highlights that aggregated analyses can identify rare variant associations that are undetectable through single-variant-based approaches, particularly for singleton and ultra-rare variants. This enhanced detection was further supported by the subgroup analysis (Supplementary Data 11), where several associations were significant only within specific ancestry. When aggregating LoF and/or deleterious missense variants, ~70% of the significant associations were specific to one ancestry group (11 out of 16 in the Bangladeshi subgroup; 7 out of 10 in the Pakistani) and did not reach cohort-wide significance. On the contrary, six (out of 13) cohort-wide significant associations did not reach significance at the individual ancestry level.

### Pleiotropy and functional context

**Fig. 5:**
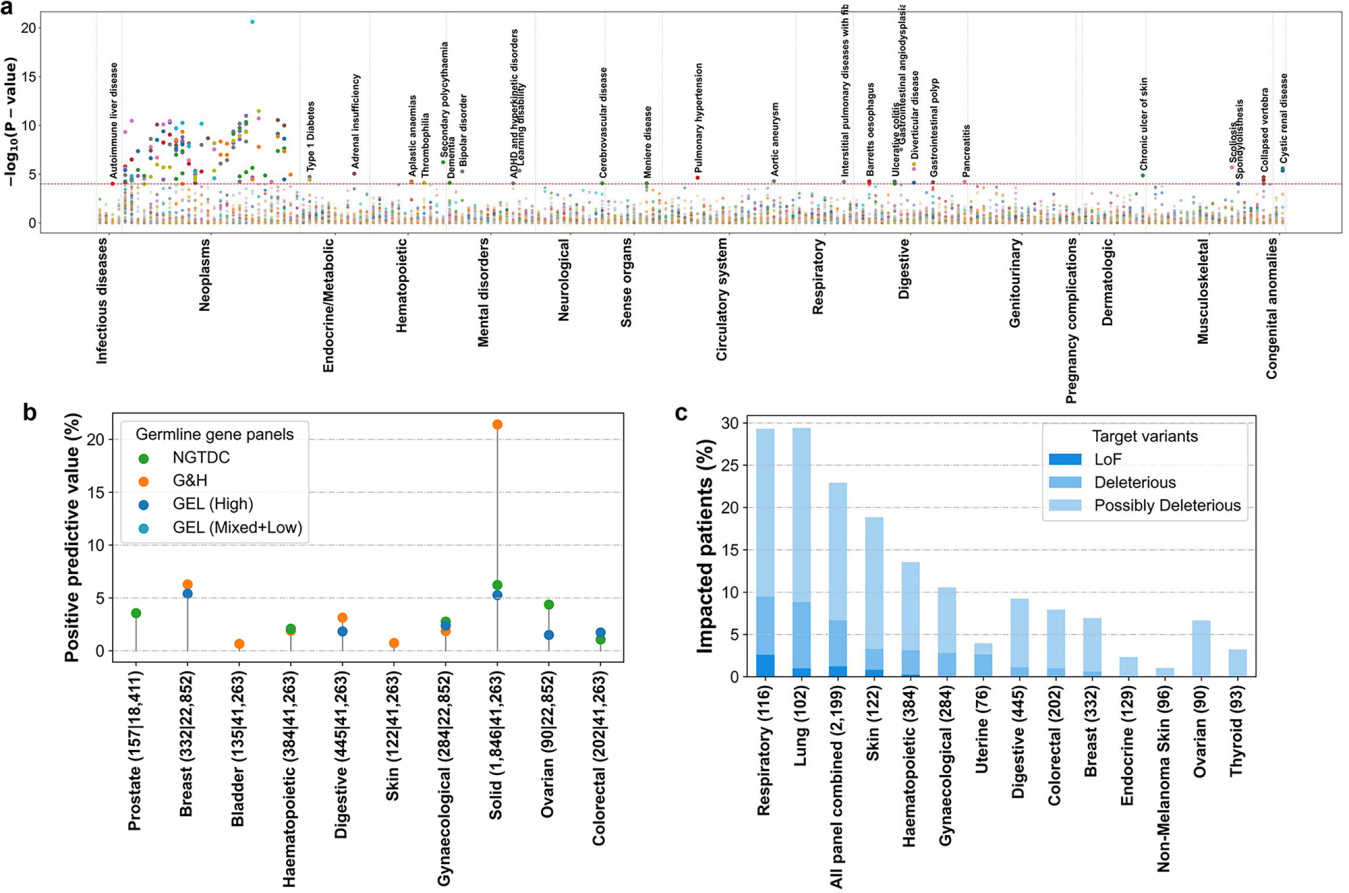
Downstream analyses of exome-wide association study results. **a** PheWAS plot showing phenome-wide associations between ExWAS-suggested variants and gene burdens (*n* = 66) and phenotypes (*n* = 187) stratified by 14 phenotype groups. Associations were tested using logistic regression under an additive genetic model; all *P-*values are two-sided. The red dashed line represents the false discovery rate significance threshold (FDR < 0.05, Benjamini–Hochberg correction; corresponding to *P* = 1.0 × 10⁻³). Only non-neoplastic phenotypes from significant associations are labelled. **b** Percentage of cancer patients who might have adverse effects or non-response to cancer drugs reported in the CGI database, grouped by cancer drug panels and variants considered. The bars are displayed in descending order of proportion of patients carrying LoF or deleterious missense variants in the target genes within a panel. Numbers in the *x*-axis represent the number of cancer patients tested. **c** Positive predictive value of known germline cancer-susceptibility gene panels (*n* = 18) along with ExWAS-suggested panels (*n* = 10) across cancer phenotypes for which LoF or deleterious missense variants were significantly enriched in participants with corresponding cancers. Numbers in the *x*-axis represent the number of cancer and non-cancer participants tested. The list of genes within each panel is provided in Supplementary Data 16.

We conducted a phenome-wide association study (PheWAS) for the 66 ExWAS-derived unique variants and gene burdens. Using a study-specific collection of 215 phenotypes, we interrogated 187 phenotypes for associations observed in at least 50 participants (see the “Methods” section). After correcting for false discovery rate (FDR < 0.05, Benjamini–Hochberg), we identified 272 significant phenome-wide associations (Fig. 5a; Supplementary Data 12). These associations were predominantly concentrated on neoplasm-specific phenotypes (*n* = 183; 67%), representing a two-fold increase over the 78 exome-wide associations observed. About 20% of the additional neoplasm-specific associations (21 out of 105) were linked to low-prevalence cancers, such as stomach cancer or CNS cancers.

Among the non-neoplastic phenotypes, digestive system diseases had the highest proportion of pleiotropic associations with cancer-susceptibility genes (20%). Comorbidity analysis revealed a significant association between gastrointestinal polyps and digestive cancers, likely driven by LoF variants in the *NF1* gene, which may serve as a useful marker for screening. Other notable pleiotropic associations included ulcerative colitis and colorectal cancer (*PDC*:p.Glu85Asp), benign CNS tumour and endocrine cancers (*H1-1*:*p*.Lys26Asn), and end-stage renal disease and urinary cancer (*CLEC16A*:*p*.Gly422Val). Additionally, we identified a pleiotropic effect of the *FKBP6* gene, which was associated with both lung cancer and various mental disorders (ADHD, bipolar disorder, schizophrenia, and psychosis). Although the involvement of other FKBP family proteins in lung cancer and mental disorders has been discussed in literature^35,36^, *FKBP6* itself has not been strongly linked to these conditions.

Among the genotypes, *CLEC16A*:*p*.Gly422Val, a possibly deleterious missense variant, had the highest number of pleiotropic associations (*n* = 16), followed by *RNF111*:*p*.His355Tyr, a deleterious missense variant, had 10 associations. Although prior reports have linked these genes or their variants to other phenotypes (Supplementary Data 8 and 10), 93% of the associations were not documented in any of the public databases queried. For example, 114 individuals (0.26%) carried at least one LoF variant in the *BRCA1* gene, with an increased risk of cancers associated with *BRCA1* mutations, including breast cancer (log-OR = 2.4 [1.6–3.3]; *P* = 6.5 × 10^−9^) and ovarian cancer (log-OR = 3.1 [2.0–4.1], *P* = 9.6 × 10^−9^)^37^. Interestingly, these individuals also exhibited an increased risk of Barrett's oesophagus (log-OR = 2.4 [1.2–3.6], *P* = 7.6 × 10^−5^), a known precursor to oesophageal cancer. This suggests an under-recognised link between pathogenic *BRCA1* mutations and oesophageal cancer^38^, especially in the context of familial neoplasms. This example highlights how our approach can uncover a broad spectrum of ancestry-specific phenotypes associated with protein-coding variations.

### Diagnostic and therapeutic implications

We evaluated the efficacy of current cancer-susceptibility gene panels in the SAS-BP population and compared them with panels derived from our study. The analysis focused on 295 unique genes across 17 cancer phenotypes: 96 genes tested clinically, as suggested by the NHS Genomic Test Directory for Cancer v11 (NGTDC)^39^; 196 genes conferring susceptibility to cancer, as reported in the Genomics England PanelApp^40^; and 54 genes significantly associated with cancer (variants collapsed at gene level) from our study (G&H).

When assessing these panels for the presence of LoF and deleterious missense variants, we identified significant enrichment of variants in 28 panels across 10 cancer types, with PPV ranging from 0.6% to 21.4% (Fig. 5b, Supplementary Data 13). Notably, G&H-derived panels demonstrated the highest PPV for 5 out of 10 cancers, including 21.4% for *solid* cancers using LoF variants, and were the only panels significantly associated with bladder and skin cancer. The overall low PPV, with only 6 out of 124 panels exceeding 5% PPV, highlights the ineffectiveness of current cancer gene panels, which were primarily developed based on evidence from European/White populations, in minority ancestry groups. This underscores the need for either ancestry-specific panels or expansion of existing panels to incorporate pan-ancestry data.

A similar challenge was observed in pharmacogenomics, where we examined the potential for tailoring cancer treatments in SAS-BP cancer patients based on their germline genomics. We found that the differentially mutated germline genes in our cohort were not significantly enriched for drug biomarkers in either a cancer-specific biomarker database (Cancer Genome Interpreter, CGI) or a broader gene–drug–disease interaction database (Pharmacogenomics Knowledgebase, PharmGKB) (Supplementary Fig. 5)^41,42^. Out of the 58 genes examined, only 2 were present in the 120 genes curated in the CGI database, and 5 were included in the 1468 for PharmGKB genes.

While somatic variants are increasingly targeted in therapeutic interventions, germline variants also play a critical role in predicting adverse drug effects or drug responses. To address this, we interrogated the CGI database to estimate the proportion of SAS-BP cancer patients who might experience adverse effects or non-response to cancer drugs based on their germline genomics (Supplementary Data 14). Among patients with LoF or deleterious variants, which are key targets in cancer pharmacogenomics, we found that up to 7% of total cancer patients could be impacted, with respiratory cancers showing the maximum impact (up to 10%). Focusing exclusively on the LoF variants, we found that current drugs could negatively affect 1% patients (Fig. 5c).

### Impact of polygenic background on exome-wide associations

To assess whether primary ExWAS associations reflected independent rare variant effects or were confounded by co-segregating common variant polygenic burden, we repeated primary ExWAS analyses for 16 cancer phenotypes with inclusion of the cancer-matched standardised polygenic risk scores (PGS) as a covariate. Effect sizes for ExWAS associations were largely unchanged after PGS adjustment (Pearson *r* = 0.99 and 0.96 for variant and gene-level association, respectively, Supplementary Fig. 6). For instance, for prostate cancer association with *ZNF155*:*p*.Glu397fs—association with the largest effect size, a 1.2% attenuation was observed (unadjusted log-OR = 7.0 [5.0–9.1], *P* = 2.8 × 10^−7^ vs. PGS-adjusted log-OR = 6.9 [4.9–8.9], *P* = 2.7 × 10^−7^). Minor discordances were observed for a small subset of gene-level associations (visible as distinct bands in Supplementary Fig. 6), characterised by singleton or ultra-rare variants aggregation (95% vs. 44% in concordant associations; 97% vs. 40% among nominally significant ones), higher estimation uncertainty (median 3.8-fold higher standard errors), and enrichment for gender-specific cancers with smaller sample sizes. These patterns indicate that the discordant bands reflect increased variability in effect size estimates due to low cumulative minor allele counts and sensitivity of rare variant aggregation tests to covariate adjustment, rather than systematic biological differences.

Across all 75 ExWAS-significant variants and gene burden, effect size estimates were largely consistent between PGS-adjusted and unadjusted models, with only modest attenuation observed, and 61 retained exome-wide significance after adjustment (Supplementary Data 15). These findings indicate that the reported rare variant-based associations are not attributable to polygenic confounding from common variants captured by the array-based PGS. Stratified analyses across PGS tertiles showed broadly similar effect estimates across polygenic risk groups (Supplementary Fig. 7), with no significant monotonic trend observed across strata (Spearman *ρ* = −0.14, *P* = 0.10) and no statistically significant variant/gene × PGS interactions detected. Although some variability in stratum-specific effect estimates was observed (median log-OR change = 1.24 among nominally significant stratified associations), this heterogeneity likely reflects reduced precision due to lower allele counts within individual strata rather than true modification of rare variant effects by polygenic background.

### Cross-ancestry validation of genetic findings

We aimed to evaluate the consistency and divergence of germline genetic signals across cancer patients of SAS-BP, SAS-nBP, and European ancestry. Whole-genome sequencing data from Genomics England Cancer cohort (*n* = 135 SAS-BP; 404 SAS-nBP; 12,980 European) were used in this analysis, focusing on coding regions. We focused our evaluation on a non-redundant set of 39 variants and 26 gene-level aggregates previously identified as germline susceptibility signals in SAS-BP individuals (Supplementary Data 8 and 10). Logistic regression models were applied to assess whether these candidate variants and genes exhibited differential mutation patterns in European or SAS-nBP cancer patients. Due to the limited sample size of the SAS-BP subgroup, analyses were performed for all cancers combined (*n* = 135), solid tumours (*n* = 125), and breast cancer (*n* = 45) only.

At the variant level, only two candidates passed the minor allele count threshold (MAC ≥ 3), but neither showed nominal significance (*P* ≤ 0.05). Gene-level aggregates were defined using three aggregation models: (M1) loss-of-function (LoF) variants, (M2) LoF plus deleterious missense variants, and (M3) LoF plus deleterious or possibly deleterious missense variants. Among 78 gene-cancer comparisons (26 genes × 3 cancers), 19 (24%) demonstrated nominally significant associations (*P* ≤ 0.05) when comparing SAS-BP and European cohorts (Supplementary Data 16). Importantly, all significant associations were in a positive direction, suggesting that the implicated genes represent germline cancer susceptibility signals enriched in South Asian populations.

When comparing SAS-BP to SAS-nBP, six associations reached nominal significance. These included *FAM166C* (M3), *SEC16A* (M2), and *TMPRSS15* (M3), all of which also showed reduced mutational burden in Europeans. This indicates that these germline susceptibility loci may be specifically enriched among Bangladeshi and Pakistani cancer patients, further highlighting the need for ancestry-tailored approaches in cancer.

## Discussion

This study provides a comprehensive genome–phenome analysis of cancer in a British South Asian population, leveraging the largest clinical history-linked exome-sequenced cohort of individuals of Bangladeshi and Pakistani ancestry. By integrating electronic health records, clinical phenotypes, and whole-exome sequencing, we identified genetic and clinical determinants of cancer risk in this underrepresented ethnic group. Our findings demonstrate that current cancer diagnostic and therapeutic paradigms, which are primarily based on European populations, may not be fully applicable to South Asian populations, underscoring the need for population-specific approaches in cancer care.

However, our work is subject to several limitations. First, the lack of external validation of our results stems, in part, from the scarcity of non-European studies that combine genetic with health record data, particularly for South Asians. The case numbers in existing cohorts, such as the UK Biobank or 100K GP, were insufficient for meaningful replication at the level of primary cancer types.

Second, despite efforts to assemble a large and representative cohort, statistical power remains limited for certain cancers due to small sample sizes (Supplementary Data 18). For the ExWAS, aggregate phenotypes and gene-level collapsing tests provided adequate power for moderate effect sizes (OR ≥ 2.5) at MAF = 1%, while site-specific cancers with fewer than 200 cases required large effects (OR > 16). At MAF < 0.1%, minimum detectable effects increased substantially, and gene-level aggregation was essential for recovering signals. For the comorbidity analysis, power was similarly constrained by case numbers, with aggregate phenotypes powered to detect OR ≥ 1.3 for common conditions (~20% prevalence) but site-specific cancers requiring OR > 2.5–3.5 for equivalent power. This should be considered when interpreting the absence of significant associations for less prevalent cancer types or low-MAF variants or low-prevalence comorbidities. The observed inverse exposure-frequency–effect relationships also reflect statistical power constraints while reinforcing biological plausibility—purifying selection maintains highly functional genetic variants at lower population frequencies, while localised pathological processes (inflammation, tissue damage) in low-prevalence organ-specific conditions produce stronger associations when cancer arises in the same organ system.

Third, while ExWAS is a powerful tool for identifying genetic variants in coding regions associated with disease risk, it captures only a fraction of the heritable risk and overlooks structural variants and epigenetic factors. Additionally, the cross-sectional nature of EHR data precludes causal inference regarding the temporal relationship between risk factors and cancer outcomes. However, the integration of pleiotropy, comorbidity, and drug-target analyses offers functional context to many of our findings.

Finally, the ancestry-specific nature of this cohort should be considered when interpreting findings. Differences in allele frequencies, linkage disequilibrium patterns, and gene–environment interactions may limit generalisability to other populations, including other South Asian subgroups. While associations involving well-established cancer genes are more likely to generalise, associations not currently represented in existing genomic resources—particularly those driven by population-specific rare variants—require independent validation. Findings may be most transferable to South Asian diaspora populations in comparable Western settings.

A key strength of our study is the enhanced accuracy of phenotype definitions through the integration of both primary and secondary care records. Unlike many phenome-wide genetic studies that rely solely on ICD-based billing codes from secondary care—an approach prone to phenotype misclassification^26^—our approach developed a more robust phecode framework by incorporating structured primary care data. This comprehensive coding strategy improves phenotypic precision and adds an additional layer of confidence to our association findings.

Our results highlight several important clinical insights. Notably, we observed an earlier age of cancer onset in South Asian individuals, particularly among Bangladeshi participants, compared to national benchmarks. This finding underscores the need to re-evaluate current age-based screening guidelines for cancers such as breast and colorectal, which may not account for earlier disease onset in this population. We also identified 147 significant cancer-phenotype associations across various organ systems, with both systemic and organ-specific comorbidities playing a prominent role in cancer predisposition. Many associations—such as the link between fibromyalgia and haematological cancers, or gastrointestinal polyps and colorectal cancer—may point to diagnostic overlap, while others, like hypertension and obesity, reinforce known modifiable risk factors that warrant targeted public health intervention in these communities.

Exome-wide analyses identified 39 rare coding variants and 31 gene-level associations with cancer phenotypes, over 60% of which were absent from major genomic databases assessed. High-confidence findings, such as *ZNF155* in prostate cancer and *NF1* in adult solid tumours, provide insights into cancer susceptibility, alongside pleiotropic variants implicated in both cancer and non-neoplastic diseases. Importantly, many of these associations were ancestry-specific and undetectable at broader cohort analyses, highlighting the relevance of substructure-aware analyses in genetically clustered populations. Several of the identified genes have plausible roles in cancer biology, supporting the functional relevance of these findings. For example, *NF1*, a negative regulator of RAS–MAPK signalling, supports a broader role as a tumour suppressor. Similarly, *ARHGAP45*, involved in cytoskeletal organisation and cell migration, aligns with pathways relevant to tumour progression, while *FKBP6*, a member of the FK506-binding protein family, may contribute through hormone or stress-response signalling. Zinc finger proteins such as *ZNF155* have been widely implicated in oncogenic processes, including cell proliferation, differentiation, and tumour progression, and provide a plausible basis for the observed prostate cancer association. In addition, the association between *BRCA1* loss-of-function variants and Barrett’s oesophagus may reflect increased susceptibility to DNA damage under chronic inflammatory conditions. More broadly, pleiotropic associations identified through PheWAS provide complementary evidence linking these loci to relevant biological pathways. While these interpretations are hypothesis-generating, they offer a biological context for the findings and highlight candidate pathways for future functional validation.

Incorporating polygenic scores into the ExWAS framework had minimal impact on the identified associations, and both stratified analyses across PGS tertiles and formal interaction testing provided no evidence that polygenic background substantially modifies rare variant effects in this cohort. Together, these findings support an additive genetic architecture for cancer risk, in which rare high-impact variants and common-variant polygenic burden contribute largely independently to overall susceptibility, consistent with the liability threshold model of complex disease^43^. From a clinical perspective, this suggests that integrated risk models incorporating both rare variants and polygenic scores may improve risk stratification, while also indicating that polygenic background does not substantially alter the penetrance of rare variants. Individuals carrying pathogenic rare variants are likely to remain at elevated risk regardless of their polygenic profile, arguing against the use of PGS to attenuate rare variant findings in clinical settings. However, interpretation should consider that most available PGS are derived from European ancestry populations and may incompletely capture the polygenic component of cancer risk in South Asian individuals, and that limited power may preclude detection of subtle interaction effects. These findings therefore support the development of ancestry-specific polygenic scores and the integration of rare and common variant information in future precision oncology frameworks for South Asian populations.

Consistent with this broader limitation in ancestry representation, current cancer susceptibility panels and pharmacogenomics resources—many of which have been developed primarily from cohorts of European ancestry—demonstrated limited predictive value in this South Asian population. Several high-penetrance gene–cancer associations identified in this study are absent from clinical testing panels, underscoring the need to diversify genomic resources and to adapt cancer screening and therapeutic frameworks to better serve underrepresented populations.

In conclusion, our work provides foundational insights into the genomic and clinical landscape of cancer in British South Asian Bangladeshi and Pakistani populations, with significant implications for ancestry-specific diagnostics, risk stratification, and therapeutic targeting. This study lays the groundwork for the inclusion of diverse populations in future cancer genomics and precision oncology efforts to ensure equitable healthcare outcomes.

## Methods

Ethical approval for the Genes & Health study was previously obtained from the Southeast London National Research Ethics Committee (14/LO/1240). Ethical approval for Genomics England 100K GP was provided by the Health Research Authority Committee East of England—Cambridge South (14/EE/1112)). Informed consent was obtained from all participants with no compensation. Data access for this research was approved by the Genes & Health Executive Board (reference: S00087) and Genomics England Clinical Interpretation Partnership (GECIP) (reference: RR1287).

### Study population

Genes & Health is a long-term, community-based study focused on British Pakistani and British Bangladeshi individuals aged 16 years and older living in the UK^27^. At recruitment, participants provide a saliva sample for germline genotyping and/or molecular sequencing, complete a short questionnaire on basic demographic information and consent to linkage with local primary care, secondary care and national-level EHRs. Since recruitment began in 2015, over 60,000 participants have been recruited. Data are available to researchers within a dedicated, trusted research environment (TRE).

### EHR data processing and curation

An initial cohort of 57,849 individuals with linked EHR data was curated from the December 2023 release of the dataset. Participant data were harmonised across primary and secondary care coding systems, including Systematised Nomenclature of Medicine (SNOMED), ICD10 (International Classification of Diseases, Tenth Revision), where applicable. The data were then organised into 216 binary phenotypes, including 38 cancer phenotypes. Each phenotype was defined by a set of ICD-10 and SNOMED codes. Participants were assigned to a phenotype if at least one corresponding clinical code was recorded in any of the four EHR sources: Discovery East London data service (East London primary care), Barts Health NHS Trust (hospital records), Bradford Teaching Hospitals NHS Foundation Trust (hospital records) and NHS Digital (Hospital Episode Statistics and Cancer Registry). The earliest recorded date of diagnosis for each phenotype was used as the diagnosis date. Participant-reported gender and ethnicity information from the questionnaire was cross-referenced with hospital records, GP data, and NHS Digital records.

### Genetic ancestry correction of ethnicity

The genome-wide genotyping data were subject to rigorous quality control procedures, as outlined by Heng et al. ^31^. Genotyping was performed using the Illumina Global Screening Array (GSAv3EAMD, build hg38) platform, and genetic ancestry (gAncestry) was inferred for each participant. Of the 51,166 genotype individuals, 51,104 were identified as having either Bangladeshi or Pakistani gAncestry. Of the remaining 6745 individuals with ambiguous gAncestry or missing genotyping data, 6312 had reported ethnicity recorded as either Bangladeshi or Pakistani. In total, 57,416 individuals with genetic ancestry-corrected ethnicity of either Bangladeshi or Pakistani were included in the analysis cohort for the comorbidity association study.

### Comorbidity association analyses

Associations between pre-existing medical conditions and cancer risk were evaluated using a generalised mixed-model framework implemented in SAIGE^44^, which accounts for both unbalanced case–control ratios and the high degree of relatedness present in the cohort. For each cancer outcome, a null model was fitted using SAIGE Step 1, including index age, index age squared, gender (excluded for gender-specific phenotypes), and gAncestry-corrected ethnicity as fixed effects and a random effect parameterised by a sparse genetic relationship matrix (GRM). The sparse GRM was constructed from pairwise kinship coefficients estimated using the KING algorithm based on genome-wide genotyping array data^45^. Only relatedness among participants up to third-degree relative (kinship coefficient ≥ 0.0442) was retained in the GRM, allowing efficient modelling of familial structure while maintaining computational scalability. This framework was used to reduce inflation of association statistics arising from familial clustering and cryptic relatedness.

Pre-existing medical conditions were treated as binary exposure variables and tested individually for association with each cancer outcome using the SAIGE Step 2 score test with saddlepoint approximation, which provides calibrated inference under case–control imbalance and relatedness. Clinical conditions were encoded as binary predictors and converted to PLINK format to enable testing within the SAIGE association framework. To ensure adequate representation of exposures within each analytical cohort, a minimum minor allele count threshold was applied to correspond to at least five individuals with the condition present.

The index date for each cancer case was defined as the diagnosis date for that cancer, regardless of whether this occurred before or after recruitment into the Genes & Health cohort and therefore included both incident and prevalent cases. The recruitment date was used as the index date for control participants. Controls for each model were selected under the following criteria: (i) no prior cancer diagnoses and (ii) exclusion of the opposite gender for analyses involving gender-specific phenotypes. The temporal relationship with pre-existing conditions was explicitly modelled: only conditions with onset on or prior to the index date were considered as exposures. Only phenotypes with a minimum of 100 cases were included in the analysis, resulting in the evaluation of 23 cancer phenotypes and 151 non-cancer phenotypes across 2782 cancer cases and 54,634 controls. The 23 cancer phenotypes were organised into a three-tier hierarchy reflecting both biological relationships and statistical considerations: primary site phenotypes (*n* = 13), representing individual cancer types defined by anatomical site; organ system phenotypes (*n* = 7), grouping cancers by shared tissue microenvironments and/or common aetiological factors; and aggregate phenotypes (*n* = 3), comprising all cancers combined, solid tumours, and secondary malignancies. This hierarchical strategy was adopted to optimise statistical power by aggregating related cancers while accommodating modest sample sizes for individual cancer types, to capture biological plausibility given that many cancer susceptibility genes confer risk across multiple cancer types, and to align with clinical utility since organ system groupings correspond to established screening and surveillance strategies.

All tests were two-sided, effect estimates were reported as odds ratios with corresponding confidence intervals, and statistical significance was considered at *P* ≤ 0.05. Multiple testing across conditions was controlled using the Bonferroni corrections. Subgroup analyses were conducted based on gender (male or female) and gAncestry-corrected ethnic subgroups (Bangladeshi or Pakistani), followed by Cochran’s Q test to identify associations with significant heterogeneity between subgroups (*P* ≤ 0.05), implying a true subgroup-specific effect.

### Whole-exome sequencing

Exome sequencing data, linked with inferred gAncestry information, were available for 44,028 participants. These data were provided in analysis-ready files in both variant call format (VCF) and PGEN format. In summary, high-depth exome sequencing was performed using the Illumina Novaseq 6000 platform (150 bp paired-end) with the Twist Clinical Research Alliance reagent at Broad Institute (Standard Germline Exome v6). Mapping to the hg38 reference genome was performed with the BWA MEM algorithm. Variants were called using the GATK HaplotypeCaller (Y chromosome genotypes were not called). The quality control (QC) process excluded samples failing sample QC, as well as variants failing a set of stringent filters (QD < 2.0, FS > 60, MQ < 30.0) and genotype quality metrics (call rate > 95%, depth > 10, quality score > 20). Due to issues with ploidy when using HaplotypeCaller, variants on chromosome X were excluded from the analysis-ready files. After removing individuals with ambiguous gAncestry or missing genotyping data, the analysis cohort was restricted to 43,462 individuals with inferred Bangladeshi or Pakistani gAncestry for subsequent ExWAS and downstream analyses.

### Variant annotation

Variants were annotated with Ensembl’s Variant Effect Predictor (VEP) v.105^46^. For each variant within a given gene, the consequence on the primary (canonical) transcript was extracted for subsequent analyses. Variants located outside the coding regions of the primary transcript, as well as monomorphic coding variants, were excluded. LoF variants were defined as those annotated as stop gained, start lost, splice donor, splice acceptor, stop lost, or frameshift, provided the allele of interest was not the ancestral allele. To assess the deleteriousness of missense variants, five annotation tools were used: SIFT^47^; PolyPhen2 HDIV and PolyPhen2 HVAR^48^; LRT^49^; and MutationTaster^50^. Missense variants were classified as *likely deleterious* if predicted as deleterious by all five tools, *possibly deleteri*ous if predicted by at least one tool and *likely benign* if not predicted to be deleterious by any tool. Subsequent analyses were restricted to LoF variants, likely deleterious and possibly deleterious variants.

### Single-variant association analyses

Association analyses were performed using the two-step exome-wide regression approach implemented in REGENIE^51^. In step 1, we included variants with MAF > 1%, with <5% genotype or sample missingness, passing the Hardy–Weinberg equilibrium test (*P* > 10^−15^) and linkage disequilibrium (LD) pruning (1000 variant windows, 100 variant sliding windows and *r*^2^  < 0.9). The choice of REGENIE as the genetic analyses tool was based on its ability to account for population structure and relatedness in step 1 through the estimation of a genome-wide polygenic effect component.

In step 2, a logistic regression model with Firth correction (for associations reaching nominal significance; *P* ≤ 0.01) was employed to test the association between each cancer type and rare variants excluding singletons. The regression models included leave-one-chromosome-out predictors obtained from step 1 as an offset, with the following covariates (i) index age, index age squared, gender, inferred gAncestry; and (ii) the first 10 ancestry-informative principal components (PCs) derived from the analysis of LD-pruned (500 variant windows, 50 variant sliding windows and *r*^2^  <  0.2) common and low-frequency variants (MAF > 1%) from the exome data. While global ancestry assignments derived from genotype array data were included as categorical covariates to account for broad population background within the Bangladeshi and Pakistani study cohort, exome-derived PCs were incorporated to adjust for fine-scale genetic structure, rare variant clustering, and technical variation within the sequenced subset used for association testing^52,53^. Concordance analyses demonstrated strong agreement across leading ancestry axes between platforms (Supplementary Fig. 8), supporting the validity of this combined framework for controlling both ancestry and pedigree structure in rare variant analyses. Only variants with a minor allele count (MAC) of five or more were analysed. We defined significant associations by a *P*-value threshold of 3 × 10^−6^, corresponding to the nominal significance level at which one positive association would be expected from the maximum number of tests (*n* = 331,291) performed for the cancer phenotype with the largest number of cases, i.e., all cancers combined (Supplementary Data 17).

### Gene-based collapsing association analyses

For gene-level association analyses, we aggregated rare variants within each gene that fit a predefined set of criteria—known as masks. Three categories of masks were considered for each gene: LoF variants (M1), LoF and likely deleterious missense variants (M2), and LoF and likely or possibly deleterious missense variants (M3). Each mask was further subdivided into four separate sub-masks based on the alternative allele frequency of the screened variants: MAF ≤ 1%, MAF ≤ 0.1%, MAF ≤ 0.01%, and singletons only. Variants that met the criteria for each mask were collapsed into a single gene-level variable by taking the maximum number of minor alleles across the sites (MAX_MA)—the default strategy in REGENIE—which treats the presence of any rare variant in the target gene as a potential risk contributor. To address the limitations of traditional burden tests, which can lose power when variant effects differ in direction, we employed the SKAT-O (Optimal sequence kernel association test) method. This test combines a burden test and a variance component test, providing greater power when variant effects oppose each other, by computing weighted averages of each statistic^54^.

The above protocol for collapsing analyses was implemented in step 2 of REGENIE, using the same set of covariates as in the single-variant analyses. To address case–control imbalance, the Firth correction was applied to calibrate the test statistics when the association reached nominal significance of *P* ≤ 0.01. A total of up to 12 SKAT-O tests (3 masks × 4 sub-masks) were performed for each potential gene–cancer association (Supplementary Fig. 9), with only the most significant association at the gene level reported. Significant associations were defined as those with *P* ≤ 6.2 × 10^−6^, corresponding to the nominal threshold at which one positive association would be expected out of the maximum number of tests (*n* = 161,671) performed for the cancer phenotype with the largest number of cases, i.e., all cancers combined (Supplementary Data 17).

### Ancestry-specific signals

We conducted both variant-level and gene-based ExWAS for individual ancestry groups, following the same protocol outlined above. Individual cancer phenotypes with at least 50 cases were included in the analysis. As a result, ovarian and uterine cancers were excluded from the Bangladeshi subgroup, while cervical, endocrine, lung, non-melanoma skin, ovarian, respiratory and uterine cancers were excluded from the Pakistani subgroup. Separate *P*-value thresholds were applied for detecting significant associations in each ancestry subgroup, set at the level where one significant association would be expected from the maximum number of tests performed. For single variant tests, the thresholds were 4.3 × 10^−6^ (Bangladeshi) and 6.3 × 10^−6^ (Pakistani). For gene-based tests, these were 7.0 × 10^−6^ and 7.5 × 10^−6^, respectively.

### Benchmarking against external resources

We assessed the overlap between our significant variant/gene–cancer associations (collapsed at the gene level) and known gene–trait associations from four sources: (i) the GWAS catalogue^55^; (ii) the OMIM database^56^; (iii) the AstraZeneca PheWAS Portal (470K WES data of European ancestry)^57^; and (iii) the FinnGen release 12^58^; all last accessed in June 2025. For the GWAs catalogue and OMIM database, we considered any variants within a gene (or in nearby regulatory/intergenic regions) that had been reported as associated with any cancer phenotype, irrespective of the variant type. We also identified genes with reported associations to other non-cancer binary phenotypes. Associations from the AstraZeneca PheWAS Portal and FinnGen were considered if reported with *P* ≤ 10^−5^. In addition, we checked South Asian ancestry-specific associations in the AstraZeneca PheWAS Portal using 500K multi-ancestry whole genome sequencing data.

### Phenome-wide association study

We used a PheWAS approach to identify the phenotypes associated with unique gene masks or single variants that reached significance across cancer phenotypes in the previous steps. For each gene mask, a modified version of the MAX_MA strategy was used to collapse qualifying variants into a binary entity representing the presence of the minor allele across the sites. We constructed a study-specific disease phecode dictionary, comprising 215 phenotypes mapped from various ICD-10 and SNOMED codes. These phecodes were curated and assigned to participants as described above. For each phecode, participants with the respective phenotype were classified as cases, while all others were considered controls. Association between each phecode and each gene mask or single variant was tested using a logistic regression model, adjusting for the same covariates as in the ExWAS. Only phecodes with at least 50 cases were included, resulting in 187 total phecodes being analysed.

### Assessment of genomic test panel genes

We tested the efficacy of cancer-susceptibility gene panels recommended for germline testing in current clinical settings. The genes selected for analysis were derived from those included in the NHS National Genomic Test Directory for Cancer (NGTDC) v11, as well as those conferring cancer susceptibility and available for testing in the Genomics England PanelApp. The latter are rated according to a traffic light system (Green, Amber and Red) indicating high, moderate or low degree of evidence of association with cancer phenotypes. The final list included 244 unique genes across 17 cancer phenotypes in our study (Supplementary Data 19).

To evaluate the relationship between a gene panel and participants’ germline status, we used Fisher’s exact test. Genes were grouped into three panels: (i) NGTDC; (ii) genes with high evidence of association from Genomics England PanelApp Green list; (iii) genes with moderate or low evidence of association in Genomics England PanelApp Amber or Red lists. A fourth panel, comprising significant genes and variants (mapped to genes) from our study, was included for comparison (labelled G&H). Participants’ germline status was categorised into three groups reflecting the increasing mutational burden from the three gene masks in the ExWAS study. For each of the 17 cancer phenotypes, we created a contingency table to compare the number of cases with mutations in at least one susceptibility gene to the number of controls with the same criteria. Positive predictive values (PPV), odds ratios (OR) and *P*-values were reported.

### Enrichment of drug targets

We assessed the enrichment of drug targets in our cancer cohort using two publicly available drug target databases.

The first list was derived from the cancer-specific biomarker database CGI (last accessed on 5 June 2025)^41^, which reports the putative effects of mutations on cancer phenotypes as ‘Responsive’, ‘Resistant’, ‘No Response’ or ‘Increased Toxicity’. This list includes drug targets for various cancers suggested by Food and Drug Administration (FDA) guidelines (*n* = 96), National Comprehensive Cancer Network (NCCN) guidelines (*n* = 34), and those reported in clinical trials (*n* = 192), case reports (*n* = 148) and pre-clinical studies (*n* = 260). For each unique (cancer × drug family × effect) tuple, we collapsed the entry at the gene level, resulting in a final list of 874 entries, spanning 27 cancer types, 134 genes, and 139 drug families (Supplementary Data 20).

The second list was derived from the PharmGKB^42^, which curates potential gene–drug and gene–disease interactions, through an extensive review of sources such as the FDA biomarker list, the Clinical Pharmacogenetics Implementation Consortium (CPIC) genes–drugs interaction list, and published literature. From the interaction table, we identified the genes that had verified associations with any drugs. Our final list included 1643 unique genes targeted by 1292 drugs (Supplementary Data 21).

We assessed the relationship between drug targets and gene–cancer associations using Fisher’s exact test. Genes were divided into two sets: (i) significantly associated single variants and gene masks mapped to the corresponding genes (*n* = 58); (ii) genes tested in the ExWAS with non-significant associations (*n* = 16,763). For each drug target list, we created a contingency table that compared the number of significant genes from our study intersecting with the drug targets against the number of non-significant genes that intersected with the list.

To estimate the proportion of patients who might experience adverse effects or non-responsiveness to therapeutic regimens, we examined drugs listed in the CGI panel reported with the effects ‘Resistant’, ‘No Response’ or ‘Increased Toxicity’. We identified patients within each cancer group who carried at least one qualifying variant in any of the target genes in the respective panel. Variants were considered qualifying if they belonged to one of the three categories: LoF, deleterious and possibly deleterious, irrespective of the allele frequency in the cohort.

### Polygenic risk adjustment and stratified analyses

To evaluate whether rare variant associations were influenced by polygenic background, we performed additional ExWAS analyses incorporating cancer-specific polygenic risk scores (PGS) as covariates. PGS were derived from previously published genome-wide models obtained from the PGS Catalogue^59^. Preference was given to scores derived from large genome-wide association studies with publicly available variant weight files. Where available, models with demonstrated applicability to South Asian populations were prioritised; otherwise, widely used models validated in large European ancestry cohorts were selected. Because most currently available polygenic risk scores have been developed primarily in populations of European ancestry, these scores were used to approximate the polygenic component of cancer risk in our cohort, recognising that predictive performance may be attenuated in non-European populations. PGS-based analyses were restricted to 16 cancer phenotypes that closely matched phenotypes available in the PGS Catalogue (Supplementary Data 22).

Rare variant and gene-level association testing was then repeated using the same analytical framework implemented in REGENIE, with PGS included as an additional covariate alongside index age, index age squared, gender, inferred genetic ancestry (gAncestry), and the first 10 exome-derived principal components. PGS were calculated from genotyping array data using the corresponding published variant weights and were standardised within the cohort prior to inclusion in the association models.

To further assess whether polygenic background modified rare variant effects, we conducted stratified analyses by dividing participants into tertiles (low, intermediate and high polygenic risk) based on the standardised PGS for each cancer phenotype. Association tests were repeated within each PGS stratum using the same modelling framework. In addition, interaction models including a variant- or gene-burden × PGS interaction term were fitted to formally test whether rare variant effects differed across the polygenic risk distribution. To preserve statistical power and reduce multiple testing, these stratified and interaction analyses were restricted to 73 unique variants and gene burdens identified in the primary ExWAS (before and after polygenic risk adjustment).

### Processing of the Genomics England dataset

The GEL 100K GP release v19 contains whole-genome germline-sequenced data for participants with cancer and rare diseases, linked to their associated clinical data, and is available to researchers within a dedicated TRE. Participants were assigned to one of the six ancestry supergroups—European, African, South Asian, East Asian, American Admix and Admix, inferred from genotyping data, using AggV2—a multi-sample genomic VCF file made from release 10 of the 100K GP project and comprises 78,195 germline genomes aligned to GRCh38 and passed quality control^60^.

For a cross-ancestry validation study, we identified 23 cancer cohorts aligned to G&H cancer phenotypes, based on primary cancer type diagnoses from the GEL core cancer dataset. In addition, the NHS England secondary dataset within GEL was interrogated for matched ICD-10 codes of 38 cancer phenotypes (see EHR data processing and curation section). For gender-specific cancers, the respective cohort was subsequently filtered to include only male or female participants as appropriate. For a more granular analysis of the South Asian population, participants were further categorised into two groups: (i) individuals identified as having Bangladeshi or Pakistani ethnicity from the Participant Summary secondary dataset (SAS-BP); (ii) the remaining individuals not of Bangladeshi or Pakistani ethnicity (SAS-nBP). This led to a validation cohort of 13,519 participants representing three groups: SAS-BP (*n* = 135), SAS-nBP (*n* = 404), and European (EUR; *n* = 12,980).

The AggV2 file was used to generate summary statistics for samples and variants annotated by VEP (v109). To compare the germline profile of SAS-BP cancer patients with respective SAS-nBP and EUR cancer patients, we performed both variant- and gene-level association analyses. Gene-level analysis was conducted by aggregating rare coding variants according to specific masks, outlined above. Individual germline genomes were passed through the GEL small variant workflow (v3.1.4) to extract the coding region variants within specific genes. Briefly, this takes the raw germline VCF files, filters on the participants and the relevant genes, annotates the variants, applies the masks, and generates a single multi-sample VCF file. For each cancer phenotype and comparison groups (SAS-BP vs. SAS-nBP, SAS-BP vs. EUR), individual logistic regression models were employed to test the association with either a single variant- or gene-level variable. The gene-level variable, defined by the given gene–mask combination, was assigned the value of the maximum number of minor alleles across the qualifying sites (MAX_MA strategy). The regression models included gender as a covariate for cancers not specific to any gender.

### Power and detectable effect sizes

We performed power analyses to estimate minimum detectable effect sizes for both the comorbidity association analyses and ExWAS, following the Wald test framework for logistic regression^61^. For case-control logistic regression, the minimum detectable log-odds ratio (β) at significance threshold α with 80% power is given by

1 $$\left|{\beta }_{\min }\right|=\left({z}_{\alpha /2}+{z}_{\beta }\right)\times {\mathrm{SE}}\left(\hat{\beta }\right)\,$$

where

2 $${\mathrm{SE}}\left(\hat{\beta }\right)=\frac{1}{\sqrt{I\left(\beta \right)}\,}$$

The Fisher information is approximated as

3 $$I\left(\beta \right)\approx \left(\frac{{n}_{1}\times {n}_{0}}{N}\right)\times 2p\left(1-p\right)$$

where *n₁* denotes the number of cases, *n*₀ the number of controls, *N* the total sample size, and *p* the exposure frequency.

For the comorbidity association analysis, power calculations used binary exposure (comorbidity present/absent), with exposure prevalence calculated as the proportion of the total study population (cases plus controls) with each pre-existing condition. For single-variant and gene-based association tests under an additive genetic model, exposure frequency was represented by MAF and cumulative MAF, respectively. Detailed power estimates stratified by cancer phenotypes and exposure frequency are provided in Supplementary Data 18.

### Reporting summary

Further information on research design is available in the Nature Portfolio Reporting Summary linked to this article.

## Supplementary information

Supplementary Information

Description of Additional Supplementary Files

Supplementary Data 1-22

Reporting Summary

Transparent Peer Review file

## Data Availability

All individual-level clinical and molecular data from Genes & Health are held securely within a trusted research environment, and direct export or public deposition of the data is not permitted to protect participant confidentiality. Instead, access is available to bona fide researchers worldwide by application to the Genes and Health Executive at https://www.genesandhealth.org/researchers/apply-for-access/. Applications are reviewed on a rolling monthly basis, and approved researchers are granted access to the individual-level data within the Trusted Research Environment throughout the duration of the bona fide research project. Genomic and phenotypic data for the 100K GP study participants are available through the Genomics England Research Environment via application at https://www.genomicsengland.co.uk/join-us: approval to access the anonymised data through the Genomics England Trusted Research Environment requires a research project proposal and mandatory training on information governance. For both Genes & Health and 100K GP studies, only processed aggregate-level data can be exported from the trusted research environments for research purposes. The aggregate-level comorbidity association data and ExWAS summary statistics that support the core findings of this study are available in Code Ocean with the identifier 10.24433/CO.1264962.v2, under a Creative Commons Attribution-NonCommercial-NoDerivatives 4.0 International licence (CC BY-NC-ND 4.0; https://creativecommons.org/licenses/by-nc-nd/4.0/). The lists of genes in genomic test gene panels, gene-drug interactions, and pharmacogenomics actionable genes that support the downstream analyses of ExWAS findings are provided in Supplementary Data 19–21. SNPs and the weights for polygenic risk scores are available in the PGS Catalogue (www.pgscatalog.org) and score IDs are provided in Supplementary Data 22. GnomAD v4.1 Exomes VCFs were obtained from https://ftp.ensembl.org/pub/data_files/homo_sapiens/GRCh38/variation_genotype/gnomad/v4.1/exomes/.

## Code availability

All software packages used to perform association analyses on individual-level data are publicly available. The comorbidity association analyses were conducted using Saige v1.3.3 (https://github.com/saigegit/SAIGE). Variant-level statistics were generated with PLINK version 2.0.0 (https://www.cog-genomics.org/plink/2.0/) and HTSlib 1.22 (https://github.com/samtools/htslib). REGENIE version 4.1.gz was used to conduct the ExWAS analyses (https://github.com/rgcgithub/regenie). The PheWAS analyses were completed using R version 4.3.3 PheWAS package (https://github.com/PheWAS/PheWAS). The downstream processing, statistical analysis and visualisation of aggregate-level data were conducted using Python version 3.9.2, available via Code Ocean at 10.24433/CO.1264962.v2, under an open-source initiative MIT licences (https://opensource.org/license/mit).
