# Supplementary material for: Genetic and health determinants of cancer risk in Bangladeshi and Pakistani individuals in the UK
Source: Nat Commun. 2026 Jun 18;17:7910. doi: 10.1038/s41467-026-74415-z (PMC13443560; doi:10.1038/s41467-026-74415-z)

**Supplementary Figure 1. Concordance between genetic ancestry and EHR-coded ethnicity for participants with genotyping array data (n= 51,166).** Genetic ancestry was inferred using principal component analysis. Concordance rate is shown on the x-axis.

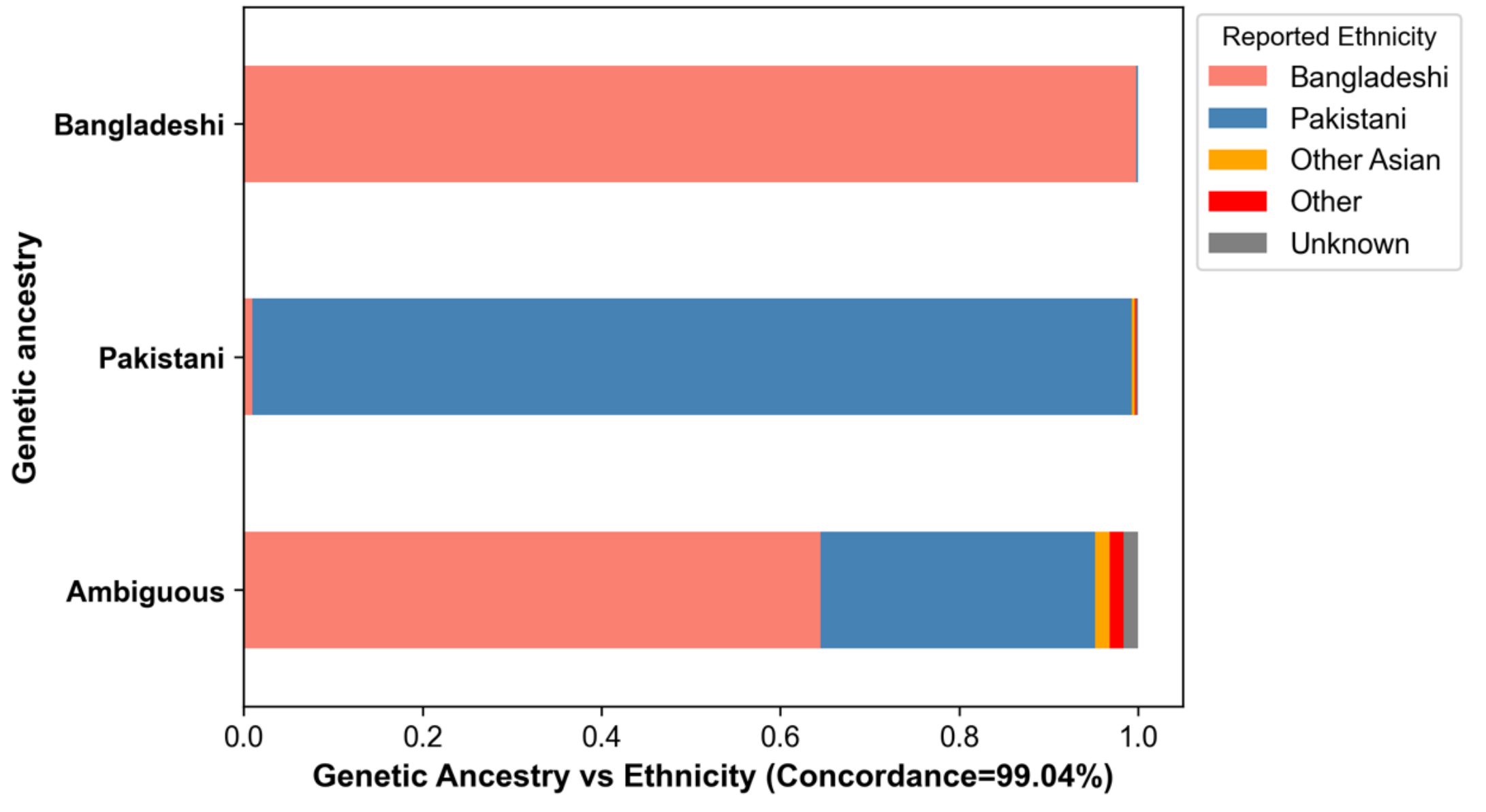


**Supplementary Figure 2. The distribution of 23 cancer phenotypes within the study (n=2,712) overlaid with participants having exome-sequenced data (n=2,199).** Phenotypes from the same organ system are grouped together. The x-axis shows the number of participants.

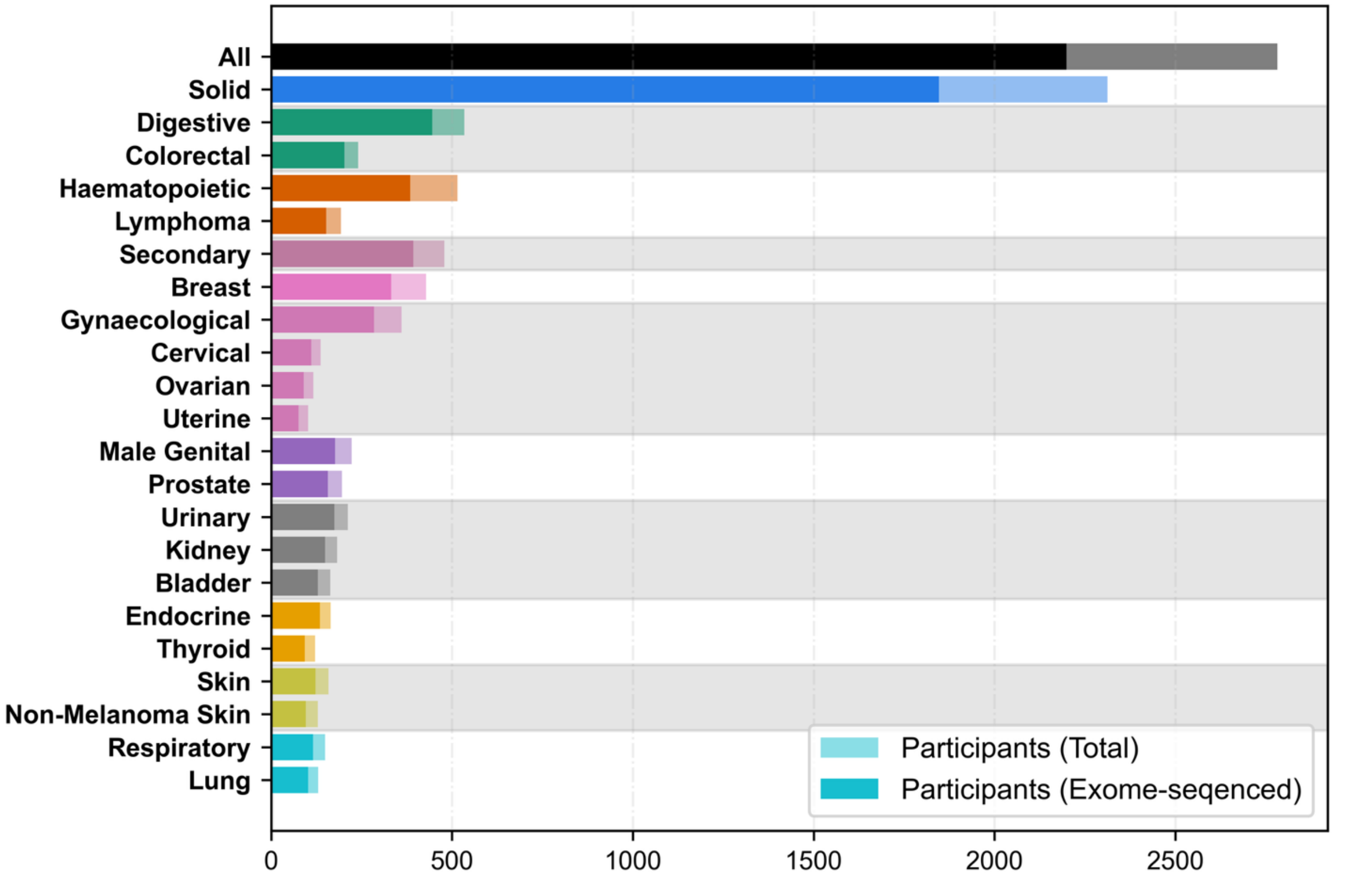

**Supplementary Figure 3. The distribution of top-20 prevalent medical conditions for each of the 23 cancer phenotypes.** Proportion of cancer patients with pre-existing medical conditions (dark green) are overlaid on proportion with lifetime prevalence (light green). The x-axis shows prevalence (%).

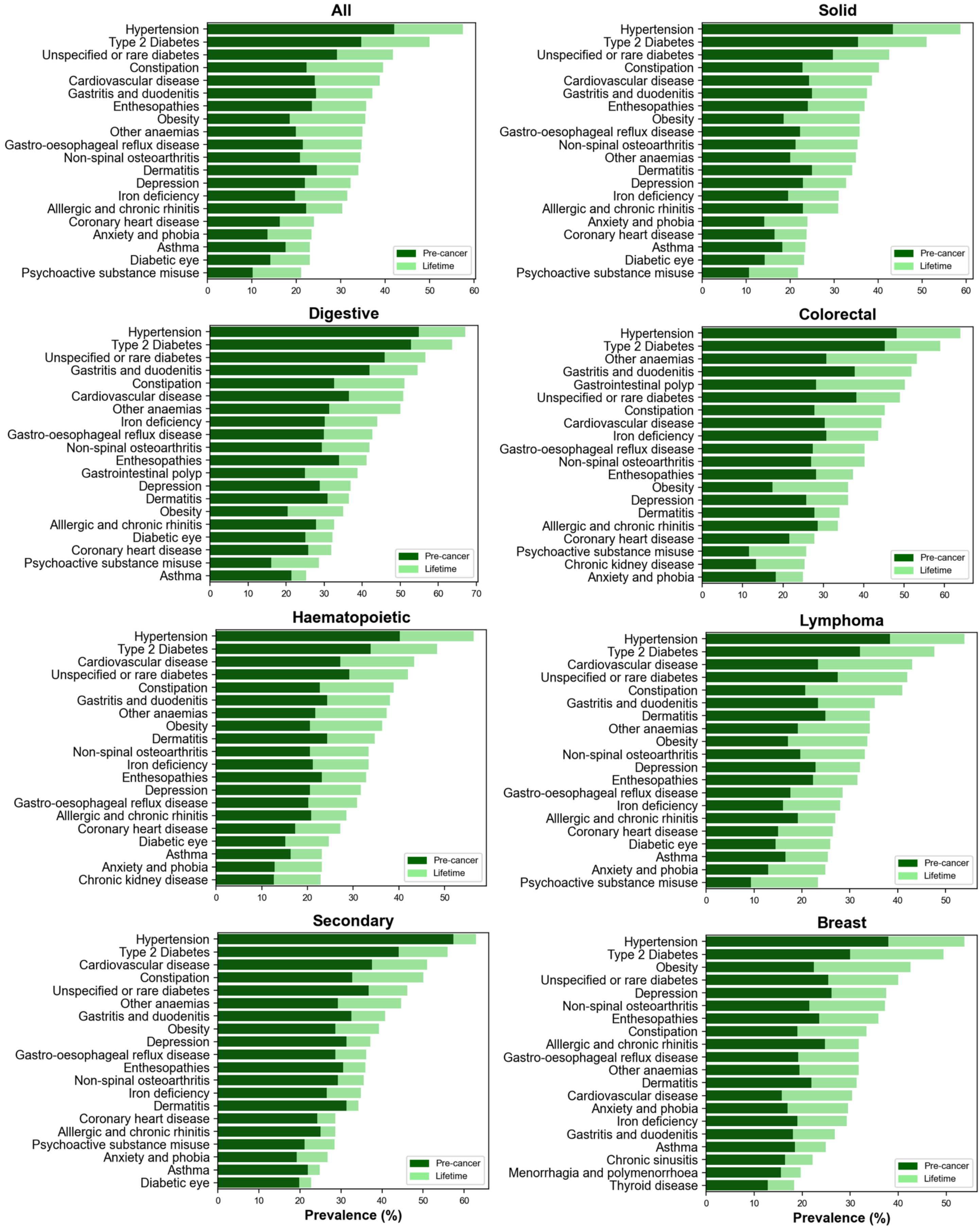

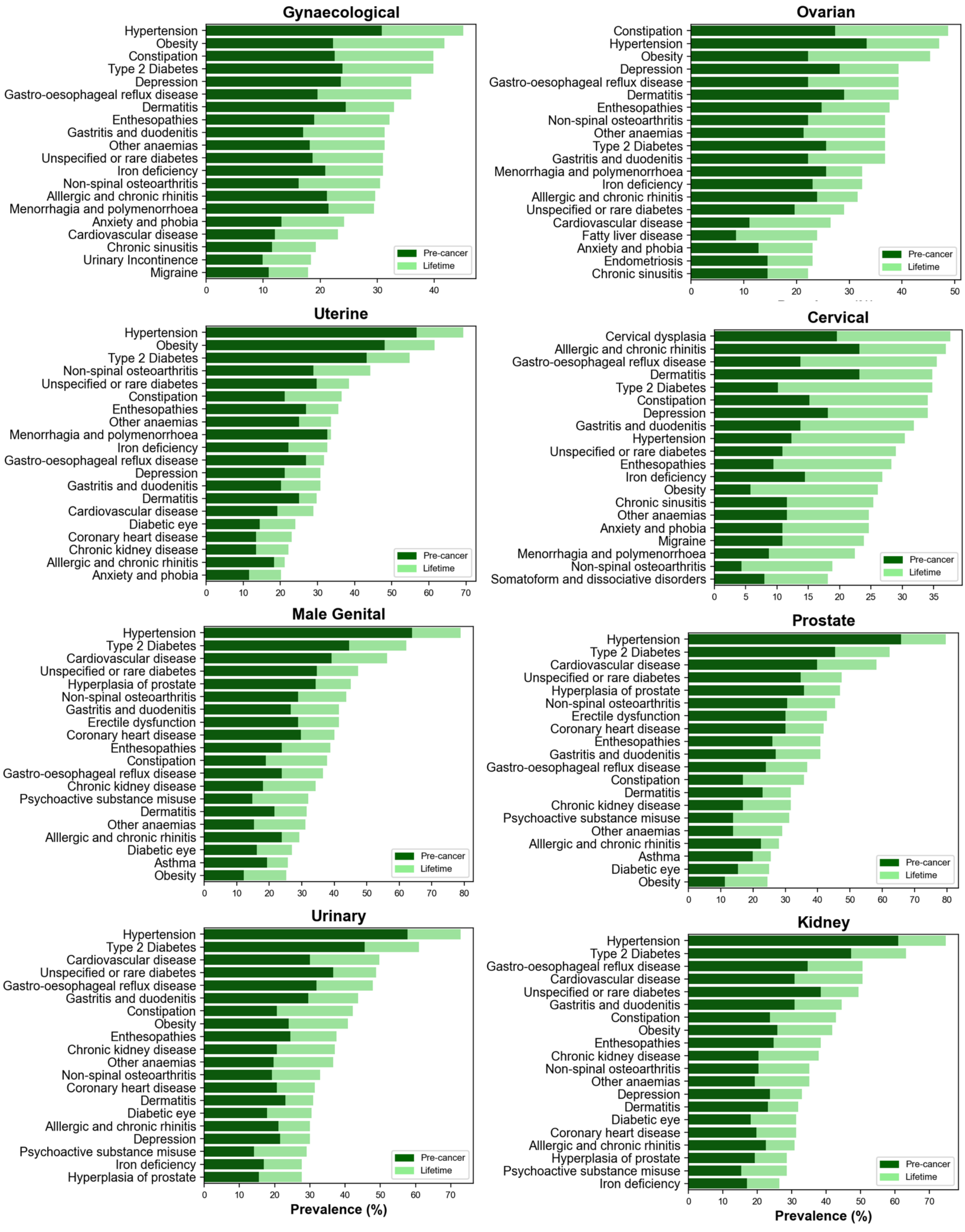

Gynaecological
Hypertension
Obesity
Constipation
Type 2 Diabetes
Depression
Gastro-oesophageal reflux disease
Dermatitis
Enthesopathies
Gastritis and duodenitis
Other anaemias
Unspecified or rare diabetes
Iron deficiency
Non-spinal osteoarthritis
Alllergic and chronic rhinitis
Menorrhagia and polymenorrhoea
Anxiety and phobia
Cardiovascular disease
Chronic sinusitis
Urinary Incontinence
Migraine
Pre-cancer
Lifetime
0 10 20 30 40
Ovarian
Constipation
Hypertension
Obesity
Depression
Gastro-oesophageal reflux disease
Dermatitis
Enthesopathies
Non-spinal osteoarthritis
Other anaemias
Type 2 Diabetes
Gastritis and duodenitis
Menorrhagia and polymenorrhoea
Iron deficiency
Alllergic and chronic rhinitis
Unspecified or rare diabetes
Cardiovascular disease
Fatty liver disease
Anxiety and phobia
Endometriosis
Chronic sinusitis
Pre-cancer
Lifetime
0 10 20 30 40 50
Uterine
Hypertension
Obesity
Type 2 Diabetes
Non-spinal osteoarthritis
Unspecified or rare diabetes
Constipation
Enthesopathies
Other anaemias
Menorrhagia and polymenorrhoea
Iron deficiency
Gastro-oesophageal reflux disease
Depression
Gastritis and duodenitis
Dermatitis
Cardiovascular disease
Diabetic eye
Coronary heart disease
Chronic kidney disease
Alllergic and chronic rhinitis
Anxiety and phobia
Pre-cancer
Lifetime
0 10 20 30 40 50 60 70
Cervical
Cervical dysplasia
Alllergic and chronic rhinitis
Gastro-oesophageal reflux disease
Dermatitis
Type 2 Diabetes
Constipation
Depression
Gastritis and duodenitis
Hypertension
Unspecified or rare diabetes
Enthesopathies
Iron deficiency
Obesity
Chronic sinusitis
Other anaemias
Anxiety and phobia
Migraine
Menorrhagia and polymenorrhoea
Non-spinal osteoarthritis
Somatoform and dissociative disorders
Pre-cancer
Lifetime
0 5 10 15 20 25 30 35
Male Genital
Hypertension
Type 2 Diabetes
Cardiovascular disease
Unspecified or rare diabetes
Hyperplasia of prostate
Non-spinal osteoarthritis
Gastritis and duodenitis
Erectile dysfunction
Coronary heart disease
Enthesopathies
Constipation
Gastro-oesophageal reflux disease
Chronic kidney disease
Psychoactive substance misuse
Dermatitis
Other anaemias
Alllergic and chronic rhinitis
Diabetic eye
Asthma
Obesity
Pre-cancer
Lifetime
0 10 20 30 40 50 60 70 80
Prostate
Hypertension
Type 2 Diabetes
Cardiovascular disease
Unspecified or rare diabetes
Hyperplasia of prostate
Non-spinal osteoarthritis
Erectile dysfunction
Coronary heart disease
Enthesopathies
Gastritis and duodenitis
Gastro-oesophageal reflux disease
Constipation
Dermatitis
Chronic kidney disease
Psychoactive substance misuse
Other anaemias
Alllergic and chronic rhinitis
Asthma
Diabetic eye
Obesity
Pre-cancer
Lifetime
0 10 20 30 40 50 60 70 80
Urinary
Hypertension
Type 2 Diabetes
Cardiovascular disease
Unspecified or rare diabetes
Gastro-oesophageal reflux disease
Gastritis and duodenitis
Constipation
Obesity
Enthesopathies
Chronic kidney disease
Other anaemias
Non-spinal osteoarthritis
Coronary heart disease
Dermatitis
Diabetic eye
Alllergic and chronic rhinitis
Depression
Psychoactive substance misuse
Iron deficiency
Hyperplasia of prostate
Pre-cancer
Lifetime
0 10 20 30 40 50 60 70
Prevalence (%)
Kidney
Hypertension
Type 2 Diabetes
Gastro-oesophageal reflux disease
Cardiovascular disease
Unspecified or rare diabetes
Gastritis and duodenitis
Constipation
Obesity
Enthesopathies
Chronic kidney disease
Non-spinal osteoarthritis
Other anaemias
Depression
Dermatitis
Diabetic eye
Coronary heart disease
Alllergic and chronic rhinitis
Hyperplasia of prostate
Psychoactive substance misuse
Iron deficiency
Pre-cancer
Lifetime
0 10 20 30 40 50 60 70
Prevalence (%)

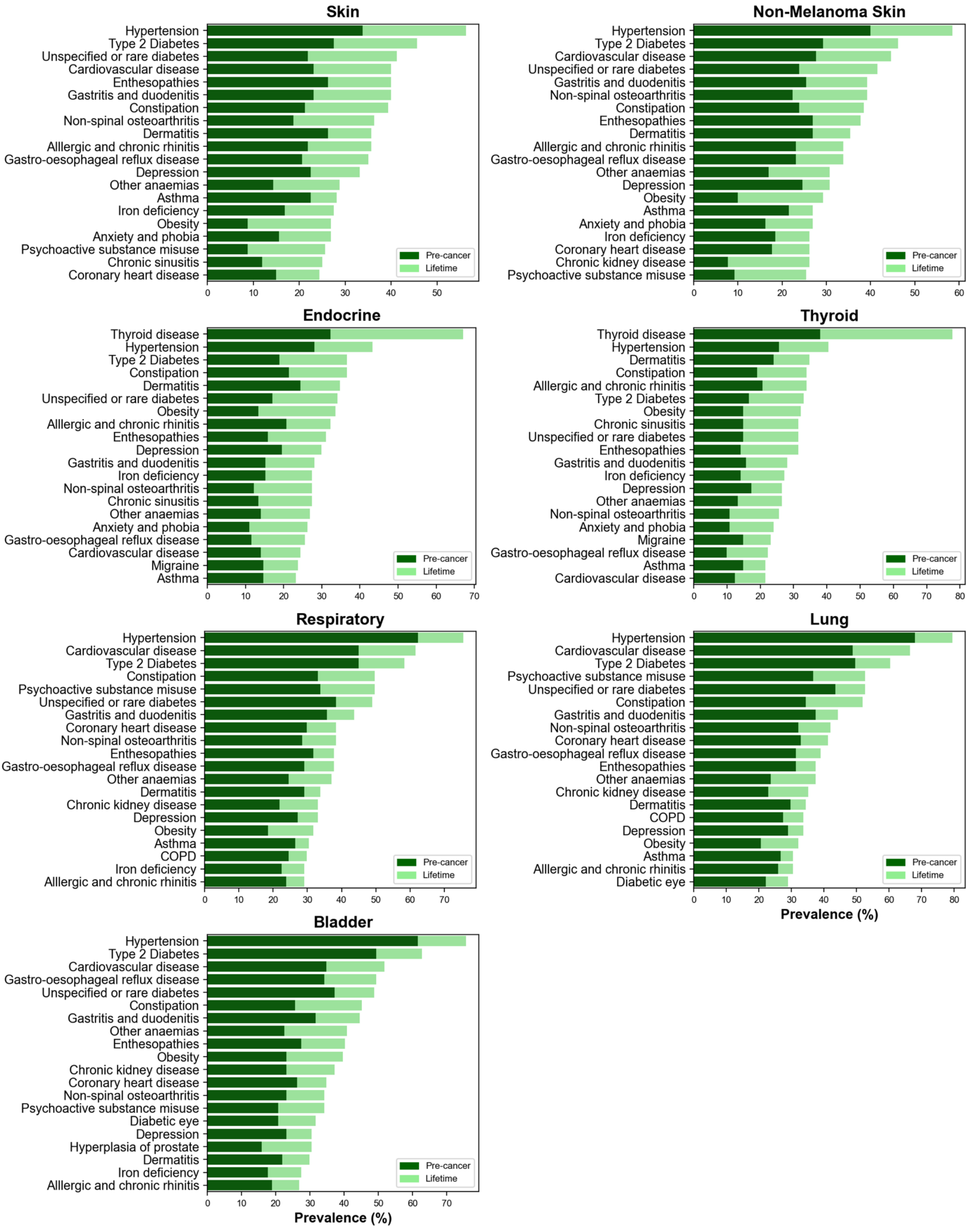
Skin
Hypertension
Type 2 Diabetes
Unspecified or rare diabetes
Cardiovascular disease
Enthesopathies
Gastritis and duodenitis
Constipation
Non-spinal osteoarthritis
Dermatitis
Alllergic and chronic rhinitis
Gastro-oesophageal reflux disease
Depression
Other anaemias
Asthma
Iron deficiency
Obesity
Anxiety and phobia
Psychoactive substance misuse
Chronic sinusitis
Coronary heart disease
Pre-cancer
Lifetime
0 10 20 30 40 50
Non-Melanoma Skin
Hypertension
Type 2 Diabetes
Cardiovascular disease
Unspecified or rare diabetes
Gastritis and duodenitis
Non-spinal osteoarthritis
Constipation
Enthesopathies
Dermatitis
Alllergic and chronic rhinitis
Gastro-oesophageal reflux disease
Other anaemias
Depression
Obesity
Asthma
Anxiety and phobia
Iron deficiency
Coronary heart disease
Chronic kidney disease
Psychoactive substance misuse
Pre-cancer
Lifetime
0 10 20 30 40 50 60
Endocrine
Thyroid disease
Hypertension
Type 2 Diabetes
Constipation
Dermatitis
Unspecified or rare diabetes
Obesity
Alllergic and chronic rhinitis
Enthesopathies
Depression
Gastritis and duodenitis
Iron deficiency
Non-spinal osteoarthritis
Chronic sinusitis
Other anaemias
Anxiety and phobia
Gastro-oesophageal reflux disease
Cardiovascular disease
Migraine
Asthma
Pre-cancer
Lifetime
0 10 20 30 40 50 60 70
Thyroid
Thyroid disease
Hypertension
Dermatitis
Constipation
Alllergic and chronic rhinitis
Type 2 Diabetes
Obesity
Chronic sinusitis
Unspecified or rare diabetes
Enthesopathies
Gastritis and duodenitis
Iron deficiency
Depression
Other anaemias
Non-spinal osteoarthritis
Anxiety and phobia
Migraine
Gastro-oesophageal reflux disease
Asthma
Cardiovascular disease
Pre-cancer
Lifetime
0 10 20 30 40 50 60 70 80
Respiratory
Hypertension
Cardiovascular disease
Type 2 Diabetes
Constipation
Psychoactive substance misuse
Unspecified or rare diabetes
Gastritis and duodenitis
Coronary heart disease
Non-spinal osteoarthritis
Enthesopathies
Gastro-oesophageal reflux disease
Other anaemias
Dermatitis
Chronic kidney disease
Depression
Obesity
Asthma
COPD
Iron deficiency
Alllergic and chronic rhinitis
Pre-cancer
Lifetime
0 10 20 30 40 50 60 70
Lung
Hypertension
Cardiovascular disease
Type 2 Diabetes
Psychoactive substance misuse
Unspecified or rare diabetes
Constipation
Gastritis and duodenitis
Non-spinal osteoarthritis
Coronary heart disease
Gastro-oesophageal reflux disease
Enthesopathies
Other anaemias
Chronic kidney disease
Dermatitis
COPD
Depression
Obesity
Asthma
Alllergic and chronic rhinitis
Diabetic eye
Pre-cancer
Lifetime
0 10 20 30 40 50 60 70 80
Prevalence (%)
Bladder
Hypertension
Type 2 Diabetes
Cardiovascular disease
Gastro-oesophageal reflux disease
Unspecified or rare diabetes
Constipation
Gastritis and duodenitis
Other anaemias
Enthesopathies
Obesity
Chronic kidney disease
Coronary heart disease
Non-spinal osteoarthritis
Psychoactive substance misuse
Diabetic eye
Depression
Hyperplasia of prostate
Dermatitis
Iron deficiency
Alllergic and chronic rhinitis
Pre-cancer
Lifetime
0 10 20 30 40 50 60 70
Prevalence (%)

**Supplementary Figure 4. Proportion of different types of variants with minor allele count ≤ 5 across 2.5 million coding variants.** LoF, Loss of function.

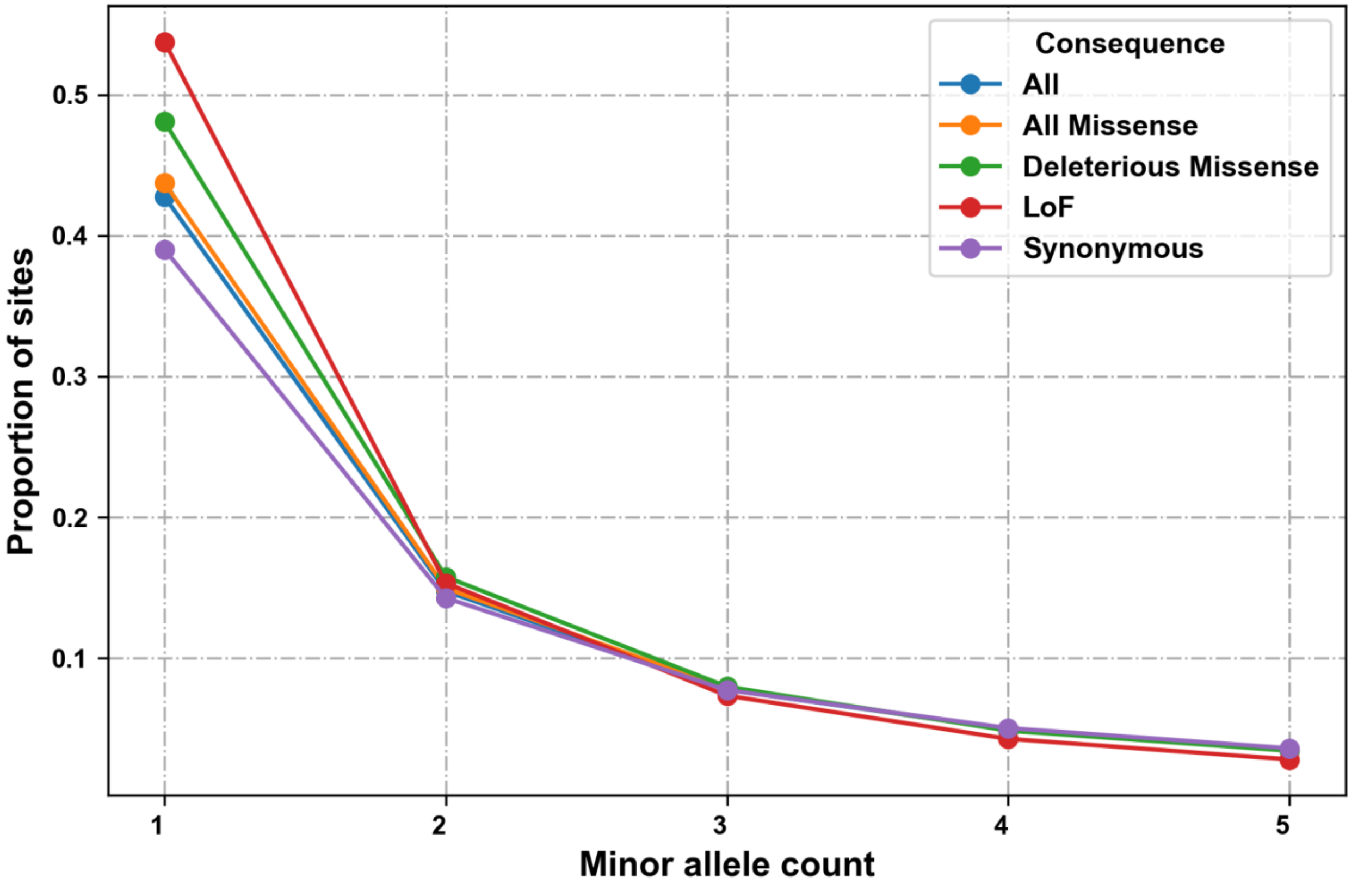


**Supplementary Figure 5. Enrichment of ExWAS-suggested genes for drugs in Cancer Genome Interpreter (CGI) and Pharmacogenomics Knowledgebase (PharmGKB) databases**. Enrichment was tested using Fisher's exact test; all P values are two-sided. Error bars represent 95% confidence intervals. The red dashed line indicates OR = 1 (no enrichment).

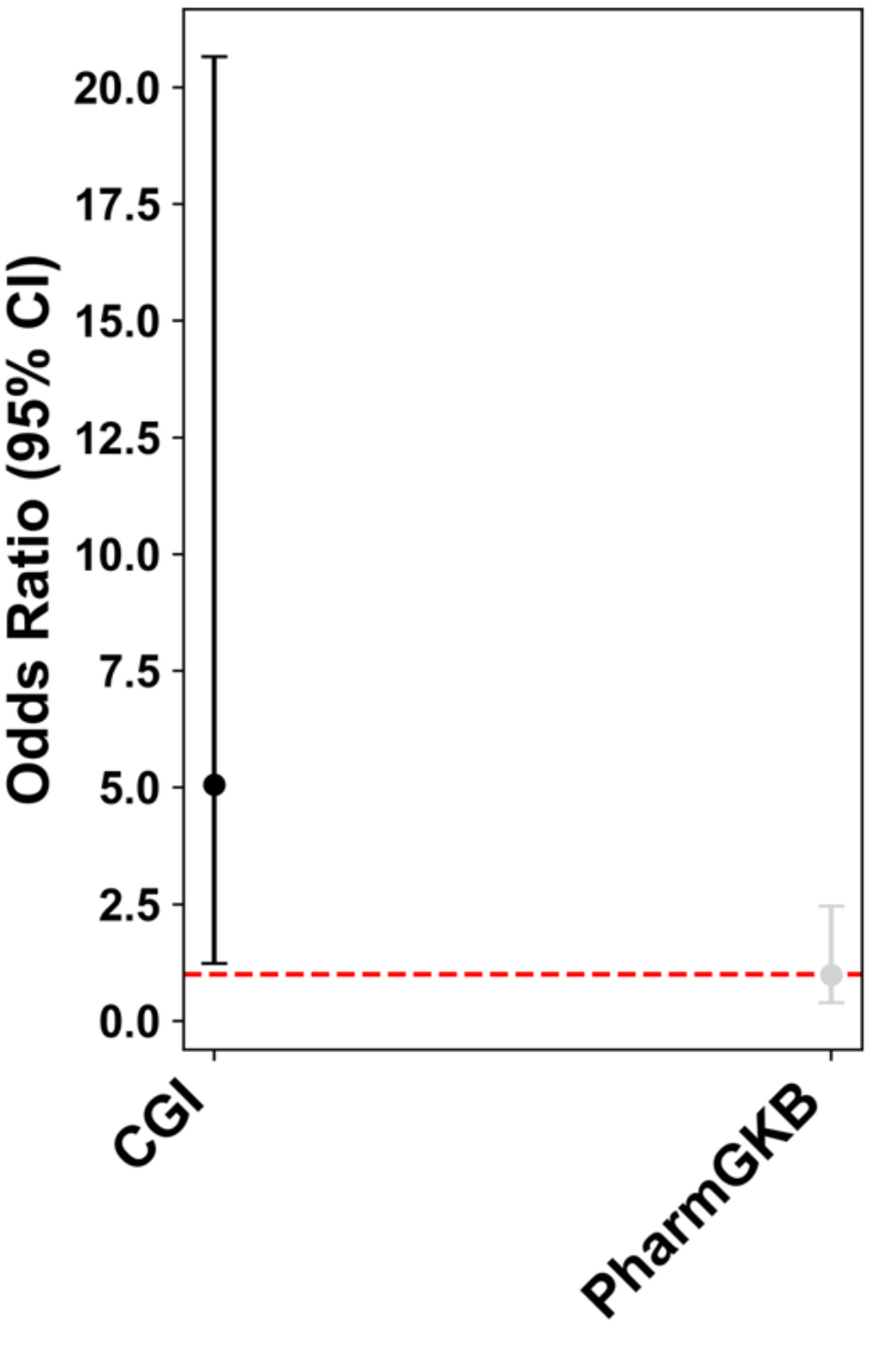

**Supplementary Figure 6. Concordance of effect sizes from primary ExWAS and PGS-adjusted ExWAS.** Each point represents a variant–cancer (left) or gene–cancer (right) association. Pearson correlation coefficients (r) are shown. The diagonal line indicates perfect concordance (y = x). PGS, polygenic risk score.

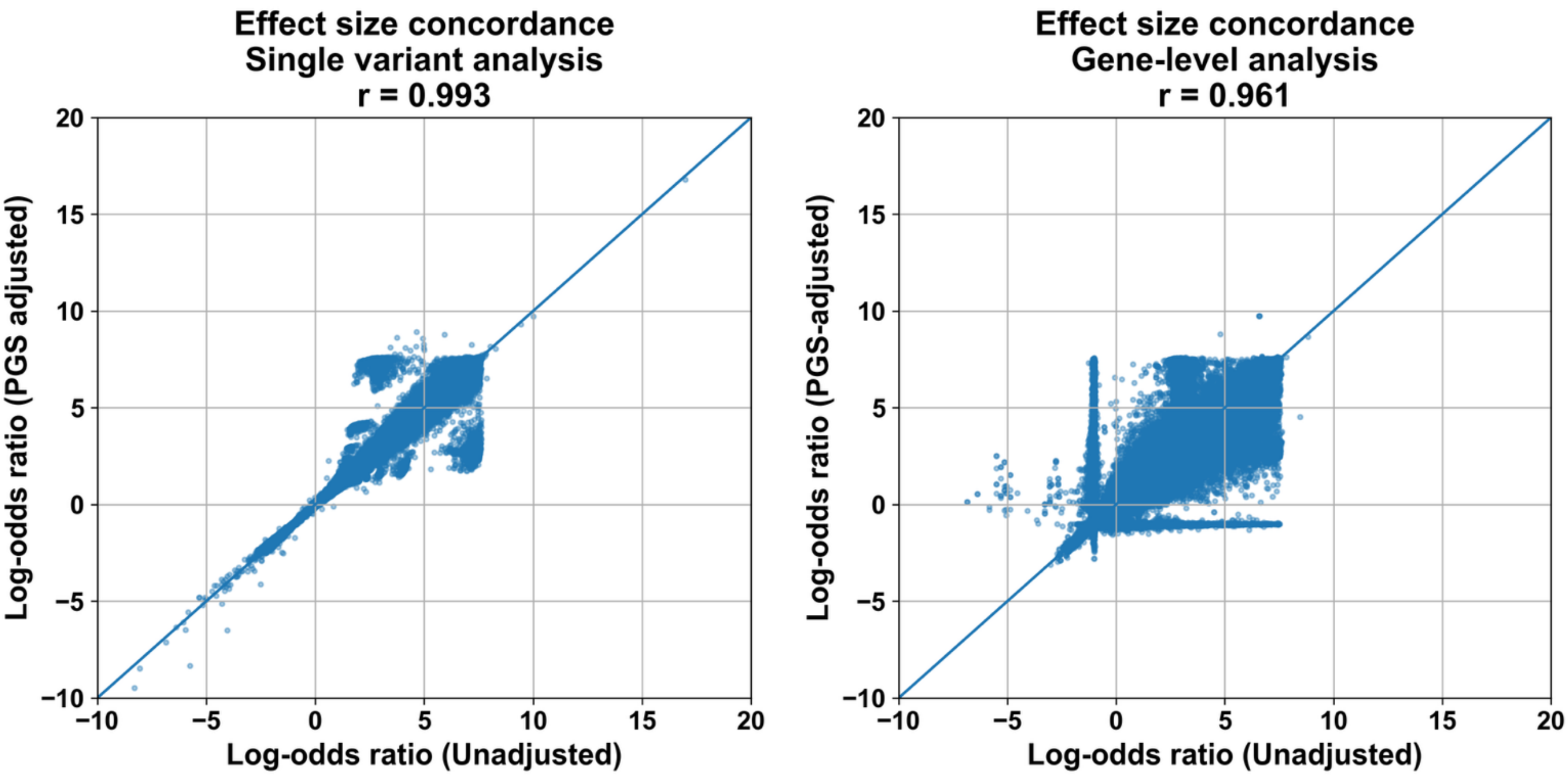

**Supplementary Figure 7. Rare variant effect sizes stratified by cancer type and polygenic risk score categories.** PGS strata are defined as Low (bottom tertile), Mid (middle tertile), and High (top tertile). Each point represents a variant or gene-burden association; labelled points reached exome-wide significance in at least one stratum. Error bars represent 95% confidence intervals. The x-axis shows log-odds ratio as effect size.

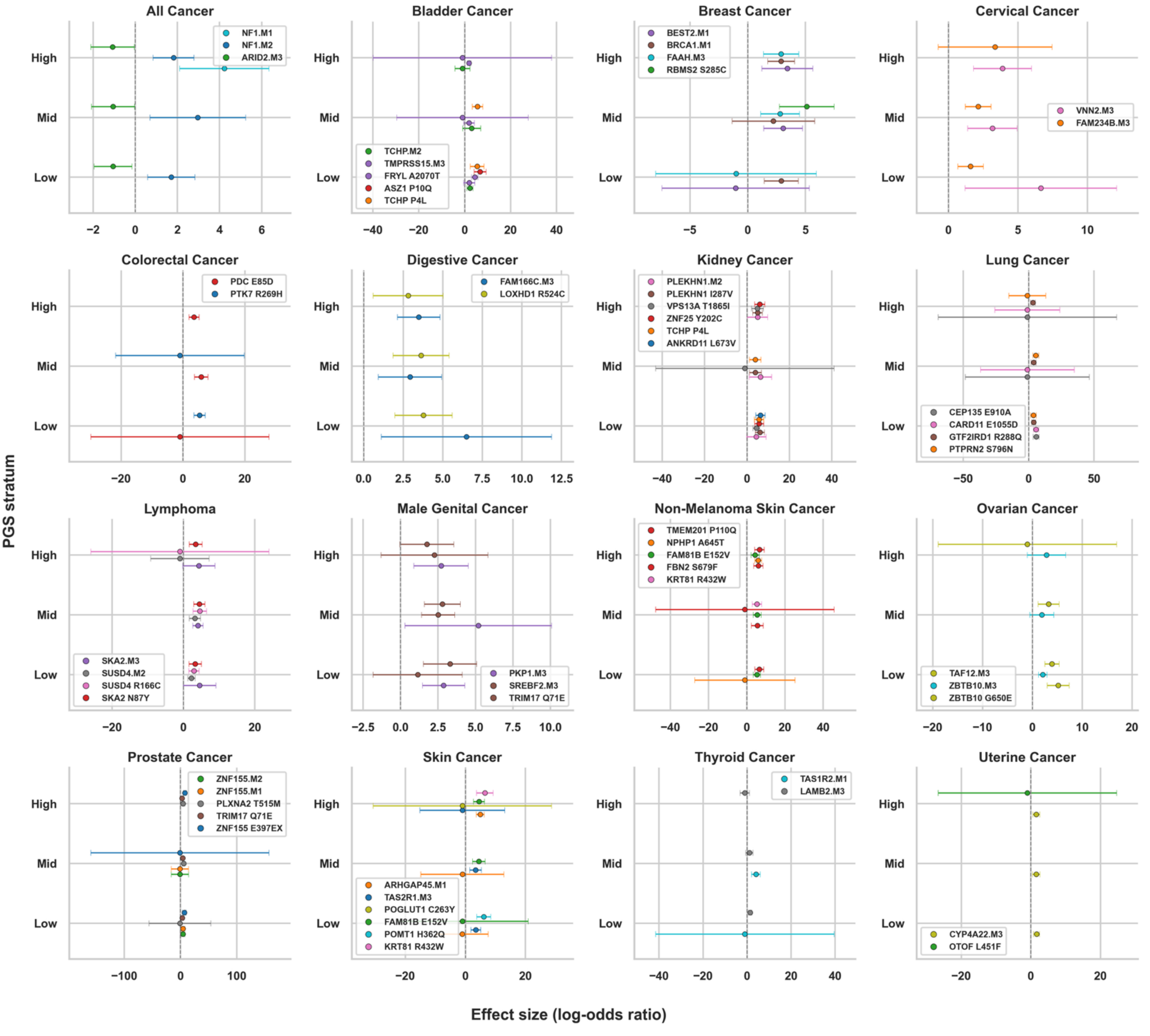

**Supplementary Figure 8. Concordance between exome- and array-derived ancestry principal components (PC1–PC3).** Each point represents a participant (n=43,462). Pearson correlation coefficients (r) are shown for each component. PC, principal component.

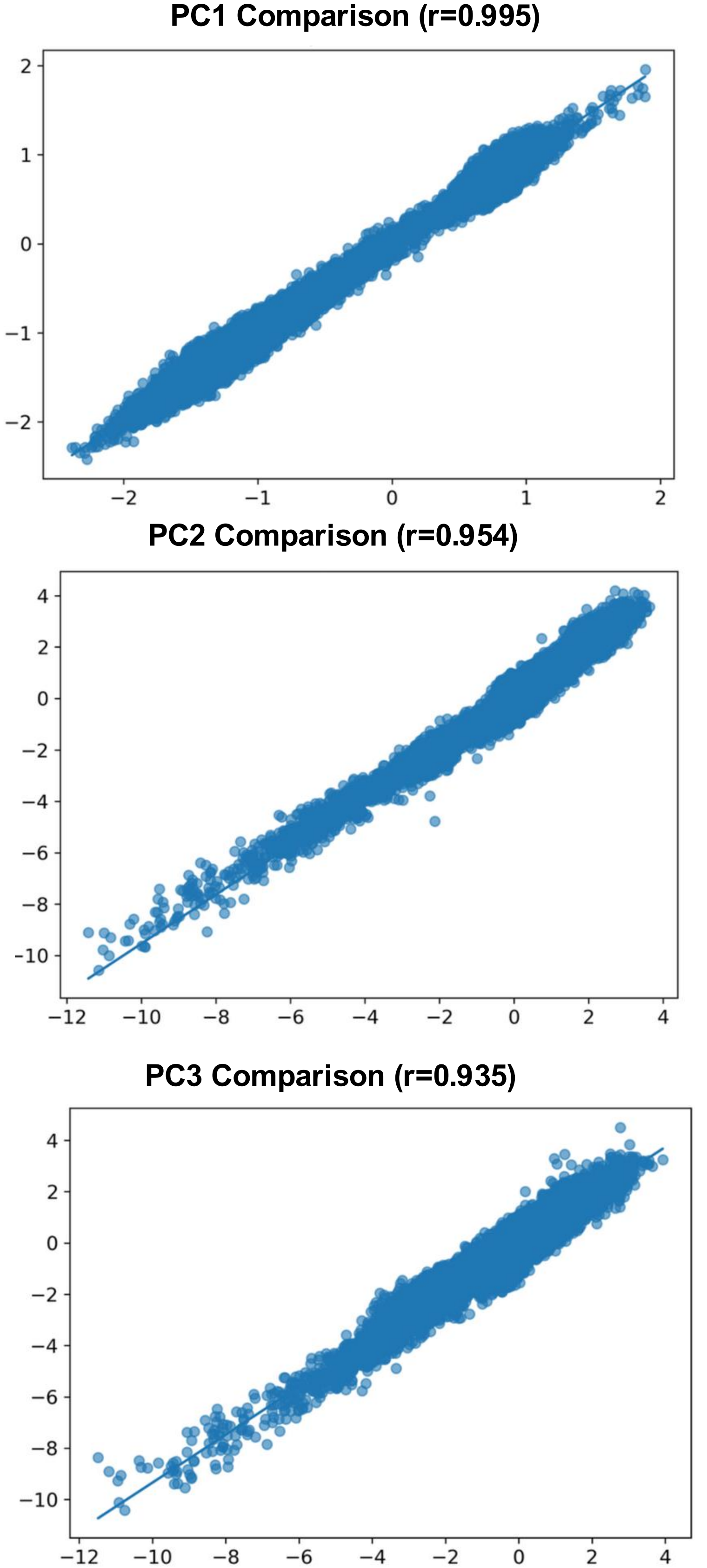

**Supplementary Figure 9. Regenie gene-based association testing method implemented in the study, illustrated for BRCA1 gene.** Three mutation burden masks (M1: LoF; M2: LoF + Deleterious; M3: LoF + Deleterious + Possibly deleterious) were tested at four alternative allele frequency (AAF) cutoffs (1%, 0.1%, 0.01%, and singletons). Association testing was performed using SKAT-O, which optimally combines burden and SKAT tests. LoF, loss-of-function; SKAT, sequence kernel association test.

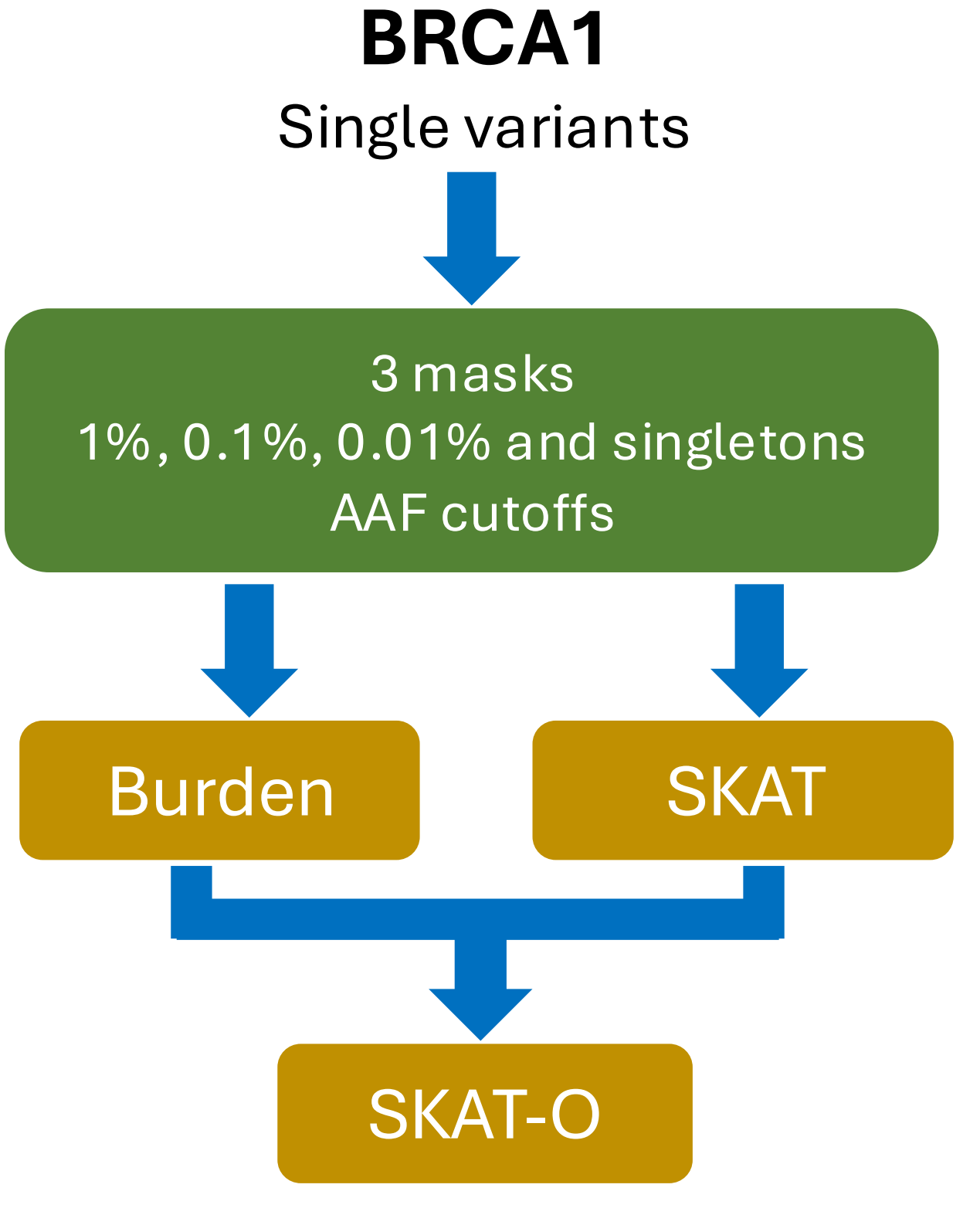

Supplement: Supplementary file 1 [file 41467_2026_74415_MOESM1_ESM.pdf]
