# Supplementary material for: Genetic and health determinants of cancer risk in Bangladeshi and Pakistani individuals in the UK
Source: Nat Commun. 2026 Jun 18;17:7910. doi: 10.1038/s41467-026-74415-z (PMC13443560; doi:10.1038/s41467-026-74415-z)
Supplement: Supplementary file 2 [file 41467_2026_74415_MOESM2_ESM.pdf]

Description of Additional Supplementary Files

File name: Supplementary Data 1

Description: Baseline characteristics of the Genes & Health **(a)** comorbidity study cohort and **(b)** whole-exome sequenced study cohort. **a,** The genetic relatedness coverage is based on the genotyping array data available for 51,104 Bangladeshi and Pakistani participants (Case, n=2,562; Control, n=48,542).

File name: Supplementary Data 2

Description: Prevalence of medical conditions in the study cohort (N=57,416). Some medical conditions are male gender specific, where prevalence is calculated across male cohort (n=25,524). Some other medical conditions are female gender specific cancer, where prevalence is calculated across female cohort (n=31,892).

File name: Supplementary Data 3

Description: Age of cancer diagnosis across different ancestries, for cancers with at least 100 cases in Genes & Health study cohort. Ancestry group with earliest diagnosis age was highlighted in bold (minimum 20 cases considered).

File name: Supplementary Data 4

Description: Associations of cancers with morbidities present before cancer diagnosis. Associations were tested using a generalised mixed-model framework (SAIGE) with score test and saddlepoint approximation. All P values are two-sided. Significance was determined using Bonferroni correction.

File name: Supplementary Data 5

Description: Associations of cancers with morbidities present before cancer diagnosis in selected demographic subgroups. Associations within gender (Male, Female) and genetic ancestry corrected ethnicity (Bangladeshi, Pakistani) subgroups were tested using a generalised mixed-model framework (SAIGE) with score test and saddlepoint approximation. All P values are two-sided. A subgroup-specific association was considered significant if it met three criteria: (1) the association survived Bonferroni correction within the subgroup; (2) only one subgroup within a category (e.g., male but not female) showed a significant association; and (3) Cochran's Q test indicated significant heterogeneity between subgroups ($P < 0.05$).

File name: Supplementary Data 6

Description: Minor allele frequency distribution across autosomal coding variants in the Genes & Health WES study cohort. This Includes a small number of coding sequence variant and incomplete terminal codon variant (n=21).

File name: Supplementary Data 7

Description: Stratified variant distribution in the Genes & Health WES study cohort and comparison with Genomics England WES cohort. **(a)** Variant distribution across cancer and control participants in the Genes & Health WES study cohort. **(b)** Variant distribution across Bangladeshi and Pakistani participants in the Genes & Health WES study cohort. **(c)** Variant distribution of cancer patients across ancestries in the Genes & Health and Genomics England WES cohorts. Genomics England study cohort only consists of those participants with germline sequencing data. **a-b,** Differences between groups evaluated by Student's t-test (two-sided).

File name: Supplementary Data 8

Description: Single variant level exome-wide significant associations with cancers in individuals of South Asian (Bangladeshi and Pakistani) ancestry. When comparing with FinnGen database, associations from LoF or missense variant tests were considered ($P < 1x10^{-5}$). When comparing with UK Biobank 470K (WES) cohort database, associations from LoF or missense single variant tests in European ancestry were considered ($P < 1x10^{-5}$). When comparing with UK Biobank 500K (WGS) cohort database, associations from LoF or missense single variant tests in South Asian ancestry were considered ($P < 1x10^{-5}$). Associations were tested using REGENIE - logistic regression under an additive genetic model. All P values are two-sided. Significant associations were defined by a P-value threshold of $3×10^{−6}$, corresponding to the nominal significance level at which one positive association would be expected from the maximum number of tests performed for *all cancer* combined phenotype.

File name: Supplementary Data 9

Description: Single variant level exome-wide significant associations with cancers in individuals of Bangladeshi and Pakistani ancestry subgroups. Associations were tested using REGENIE - logistic regression under an additive genetic model. All P values are two-sided. For each genetic ancestry subgroup, significant associations were defined by a P-value threshold of $4.3×10^{−6}$ (Bangladeshi) and $6.3×10^{−6}$ (Pakistani), corresponding to the nominal significance level at which one positive association would be expected from the maximum number of tests performed for *all cancer* combined phenotype.

File name: Supplementary Data 10

Description: Gene level exome-wide significant associations with cancers in individuals of South Asian (Bangladeshi and Pakistani) ancestry. When comparing with FinnGen database, associations from LoF gene-burden tests were considered ($P < 1x10^{-5}$). When comparing with UK Biobank 470K (WES) cohort database, associations from LoF or missense gene-burden tests in European ancestry were considered ($P < 1x10^{-5}$). When comparing with UK Biobank 500K (WGS) cohort database, associations from LoF or missense gene-burden tests in South Asian ancestry were considered ($P < 1x10^{-5}$). Associations were tested using REGENIE - SKAT-O burden test. A total of up to 12 SKAT-O tests (3 masks x 4 sub-masks) were performed for each potential gene–cancer association. Masks are categorised as LoF variants (M1), LoF and likely deleterious missense variants (M2), and LoF and likely or possibly deleterious missense variants (M3). Each mask was subdivided into four separate sub-masks based on the minor allele frequency of the screened variants: MAF ≤ 1%, MAF ≤ 0.1%, MAF ≤ 0.01%, and singletons only. All P values are two-sided. Significant associations were defined by a P-value threshold of $6.2×10^{−6}$, corresponding to the nominal significance level at which one positive association would be expected from the maximum number of tests performed for *all cancer* combined phenotype.

File name: Supplementary Data 11

Description: Gene level exome-wide significant associations with cancers in individuals of Bangladeshi and Pakistani ancestry subgroups. Associations were tested using REGENIE - SKAT-O burden test. A total of up to 12 SKAT-O tests (3 masks x 4 sub-masks) were performed for each potential gene–cancer association. Masks are categorised as LoF variants (M1), LoF and likely deleterious missense variants (M2), and LoF and likely or possibly deleterious missense variants (M3). Each mask was subdivided into four separate sub-masks based on the minor allele frequency of the screened variants: MAF ≤ 1%, MAF ≤ 0.1%, MAF ≤ 0.01%, and singletons only. All P values are two-sided. For each genetic ancestry subgroup, significant associations were defined by a P-value threshold of 7.0×10^{-6} (Bangladeshi) and 7.5×10^{-6} (Pakistani), corresponding to the nominal significance level at which one positive association would be expected from the maximum number of tests performed for *all cancer* combined phenotype.

File name: Supplementary Data 12

Description: Phenome-wide significant associations observed for 66 cancer-associated markers. When comparing with FinnGen database, associations from LoF or missense variant tests were considered ($P < 1\times10^{-5}$). When comparing with UK Biobank 470K (WES) cohort database, associations in European ancestry were considered for LoF or missense coding variants ($P < 1\times10^{-5}$) - single variant tests for single variant marker, gene-burden tests for variant aggregate marker. When comparing with UK Biobank 500K (WGS) cohort database associations in South Asian ancestry were considered for LoF or missense coding variants ($P < 1\times10^{-5}$) - single variant tests for single variant marker, gene-burden tests for variant aggregate marker. Associations were tested using logistic regression under an additive genetic model. All P values are two-sided. Significance was determined using false discovery rate correction (FDR < 0.05, Benjamini–Hochberg).

File name: Supplementary Data 13

Description: Enrichment of cancer-susceptibility panel genes in the WES study cohort. Enrichment of cancer-susceptibility panel genes was evaluated using Fisher's exact test; all P values are two-sided. Genes were grouped into four panels: (1) G&H — significant genes and variants from the current Genes & Health study; (2) NGTDC — genes from the NHS National Genomic Test Directory for Cancer v11; (3) GEL (High) — genes with high evidence of association from Genomics England PanelApp; (4) GEL (Mixed+Low) — genes with moderate or low evidence of association from Genomics England PanelApp. Participants' germline status was categorised into three groups reflecting increasing mutational burden: LoF (loss-of-function only), LoF + Deleterious, and LoF + Deleterious + Possibly deleterious. For each cancer phenotype, a contingency table compared the number of cases with mutations in at least one susceptibility gene to controls with the same criteria.

File name: Supplementary Data 14

Description: Potential impact of known cancer drugs on Genes & Health participants.

File name: Supplementary Data 15

Description: Exome-wide significant associations with cancers in individuals of South Asian (Bangladeshi and Pakistani) ancestry before and after adjusting for cancer-specific polygenic risk score. Single-variant associations were tested using REGENIE with logistic regression under an additive genetic model. Gene-level associations were tested using REGENIE - SKAT-O burden test. All P values

are two-sided. PGS-adjusted models included cancer-specific polygenic risk scores as additional covariates alongside age, age-squared, gender, genetic ancestry, and the first ten exome-derived principal components. The same significance thresholds used for identifying associations in unadjusted models were applied to adjusted models (single-variant: $P \leq 3\times10^{-6}$; gene-level: $P \leq 6.2\times10^{-6}$). All associations reaching significance are presented.

File name: Supplementary Data 16

Description: Nominally significant associations between genes and of South Asian (Bangladeshi and Pakistani) individuals compared to European and South Asian (not Bangladeshi or Pakistani) individuals. Associations were tested using logistic regression. A total of up to 3 tests were performed for each potential gene–cancer association, representing different variant aggregation masks at MAF ≤ 1%: LoF variants, LoF and likely deleterious missense variants, and LoF and likely or possibly deleterious missense variants. All P values are two-sided. All associations reaching nominal significance ($P \leq 0.05$) are presented.

File name: Supplementary Data 17

Description: Number of exome-wide association tests performed for 22 cancer phenotypes. The total number of tests performed is less than total number of possible tests (N phenotypes x N markers) because minor allele count (MAC) was <5 for some phenotype-marker pairs. For gene-based association tests, multiple masks tested per gene-phenotype pair: Singletons, MAF < 0.01%, MAF < 0.1% and MAF < 1%. Masks are only tested for association when MAC > 5.

File name: Supplementary Data 18

Description: Minimum detectable effect sizes in the study. **(a)** Minimum detectable odds ratios (OR) for comorbidity association analyses. Values represent minimum detectable odds ratio with 80% statistical power and significance threshold $\alpha = 1.45\times10^{-5}$ in two-sided tests. **(b)** Minimum detectable effect sizes (log-OR) for ExWAS analyses. Values represent minimum detectable log-odds ratio (β) with 80% statistical power in two-sided tests. '>6.9' indicates that only extremely large effects (OR > 999) would be detectable.

File name: Supplementary Data 19

Description: Genomic test panels from Genomics England PanelApp (GEL), National Genomic Test Directory for Cancer (NGTDC), and this study (G&H).

File name: Supplementary Data 20

Description: Gene-drug interaction list from Cancer Genome Interpreter.

File name: Supplementary Data 21

Description: List of actionable genes from Pharmacogenomics Knowledgebase.

File name: Supplementary Data 22

Description: Polygenic risk scores used for polygenic background adjustment in ExWAS.
