# Supplementary material for: Genetic and health determinants of cancer risk in Bangladeshi and Pakistani individuals in the UK
Source: Nat Commun. 2026 Jun 18;17:7910. doi: 10.1038/s41467-026-74415-z (PMC13443560; doi:10.1038/s41467-026-74415-z)
Supplement: Supplementary file 3 [file 41467_2026_74415_MOESM4_ESM.pdf]

nature portfolio

Corresponding author(s): Claude Chelala, Abu ZM Dayem Ullah

Last updated by author(s): May 26, 2026

Reporting Summary

Nature Portfolio wishes to improve the reproducibility of the work that we publish. This form provides structure for consistency and transparency in reporting. For further information on Nature Portfolio policies, see our Editorial Policies and the Editorial Policy Checklist.

Statistics

For all statistical analyses, confirm that the following items are present in the figure legend, table legend, main text, or Methods section.

n/a	Confirmed	
☐	☒	The exact sample size (*n*) for each experimental group/condition, given as a discrete number and unit of measurement
☐	☒	A statement on whether measurements were taken from distinct samples or whether the same sample was measured repeatedly
☐	☒	The statistical test(s) used AND whether they are one- or two-sided<br>*Only common tests should be described solely by name; describe more complex techniques in the Methods section.*
☐	☒	A description of all covariates tested
☐	☒	A description of any assumptions or corrections, such as tests of normality and adjustment for multiple comparisons
☐	☒	A full description of the statistical parameters including central tendency (e.g. means) or other basic estimates (e.g. regression coefficient) AND variation (e.g. standard deviation) or associated estimates of uncertainty (e.g. confidence intervals)
☐	☒	For null hypothesis testing, the test statistic (e.g. *F*, *t*, *r*) with confidence intervals, effect sizes, degrees of freedom and *P* value noted<br>*Give P values as exact values whenever suitable.*
☒	☐	For Bayesian analysis, information on the choice of priors and Markov chain Monte Carlo settings
☒	☐	For hierarchical and complex designs, identification of the appropriate level for tests and full reporting of outcomes
☐	☒	Estimates of effect sizes (e.g. Cohen's *d*, Pearson's *r*), indicating how they were calculated

Our web collection on statistics for biologists contains articles on many of the points above.

Software and code

Policy information about availability of computer code

Data collection	Python v3.9.2<br>PLINK v2.0.0<br>htslib v1.18
Data analysis	Python v3.9.2<br>R v4.3.3<br>Regenie v4.1.gz<br>SAIGE v1.3.3.<br>https://doi.org/10.24433/CO.1264962.v2

For manuscripts utilizing custom algorithms or software that are central to the research but not yet described in published literature, software must be made available to editors and reviewers. We strongly encourage code deposition in a community repository (e.g. GitHub). See the Nature Portfolio guidelines for submitting code & software for further information.

Data

Policy information about availability of data

All manuscripts must include a data availability statement. This statement should provide the following information, where applicable:

- Accession codes, unique identifiers, or web links for publicly available datasets
- A description of any restrictions on data availability
- For clinical datasets or third party data, please ensure that the statement adheres to our policy

All individual-level clinical and molecular data from Genes & Health are held securely within a trusted research environment, and direct export or public deposition of the data is not permitted to protect participant confidentiality. Instead, access is available to bona fide researchers worldwide by application to the Genes and Health Executive at https://www.genesandhealth.org/researchers/apply-for-access/. Applications are reviewed on a rolling monthly basis, and approved researchers are granted access to the individual-level data within the Trusted Research Environment throughout the duration of the bona fide research project. Genomic and phenotypic data for the 100K GP study participants are available through the Genomics England Research Environment via application at https://www.genomicsengland.co.uk/join-us: approval to access the anonymised data through the Genomics England Trusted Research Environment requires a research project proposal, and mandatory training on information governance. For both Genes & Health and 100K GP studies, only processed aggregate-level data can be exported from the trusted research environments for research purposes. The aggregate-level comorbidity association data and ExWAS summary statistics that support the core findings of this study are available in Code Ocean with the identifier https://doi.org/10.24433/CO.1264962.v2, under a Creative Commons Attribution-NonCommercial-NoDerivatives 4.0 International licence (CC BY-NC-ND 4.0; https://creativecommons.org/licenses/by-nc-nd/4.0/). The lists of genes in genomic test gene panels, gene-drug interactions, and pharmacogenomics actionable genes that support the downstream analyses of ExWAS findings are provided in Supplementary Tables 19-21. SNPs and the weights for polygenic risk scores are available in the PGS Catalog (www.pgscatalog.org) and score IDs are provided in Supplementary Table 22. GnomAD v4.1 Exomes VCFs were obtained from https://ftp.ensembl.org/pub/data_files/homo_sapiens/GRCh38/variation_genotype/gnomad/v4.1/exomes/.

Research involving human participants, their data, or biological material

Policy information about studies with human participants or human data. See also policy information about sex, gender (identity/presentation), and sexual orientation and race, ethnicity and racism.

Reporting on sex and gender

Self-reported gender information was collected from the primary Genes & Health participant questionnaire sheet, and triangulated with hospital records, GP data and NHS Digital records. The final dataset included 55.5% female and 44.5% male gender participants. No participants had 'unknown' or 'not available/stated' gender status .

Reporting on race, ethnicity, or other socially relevant groupings

For the primary Genes & Health dataset, reported ethnicity of Bangladeshi and Pakistani were triangulated through participant questionnaire sheets, hospital records, GP data and NHS Digital records. For 89% of these participants, genotyping array data were used to confirm their Bangladeshi and Pakistani genetic ancestry, and the reported ethnicity was corrected if necessary. For those without genetic ancestry information, the reported ethnicity was kept. At the end, only participants with genetic ancestry-corrected ethnicity of Bangladeshi or Pakistani were used in the study.

For the Genomics England 100K GP dataset, genotyping array-derived genetic ancestry was considered as true representation of European and South Asian ethnic groups. South Asian group was further categorised into 'Bangladeshi and Pakistani' and 'not Bangladeshi and Pakistani' subgroup based on reported ethnicity.

Population characteristics

The primary population of this study comprised British Pakistani and British Bangladeshi individuals aged 16years and older living in the UK, predominantly in East London, recruited by Genes & Health study. 4.8% of the population had cancers recorded in their health record, who represented the target cases for the analysis. The rest 95.2% of the population served as non-cancer controls.

The secondary dataset from Genomics England 100K GP dataset included cancer patients of European and South Asian genetic ancestry (n=13,573).

Recruitment

All recruitment was performed by the relevant study according to their protocol (Genes & Health, Genomics England 100K GP)

Ethics oversight

Ethical approval for the Genes & Health study was provided by the SouthEast London National Research Ethics Committee (14/LO/1240). An individual application to support data access for this study was granted by Genes & Health Executive Board (reference S00087).
Genomics England Research Network gave approval to support access to 100K GP dataset for this study (reference 643).

Note that full information on the approval of the study protocol must also be provided in the manuscript.

Field-specific reporting

Please select the one below that is the best fit for your research. If you are not sure, read the appropriate sections before making your selection.

☒ Life sciences ☐ Behavioural & social sciences ☐ Ecological, evolutionary & environmental sciences

For a reference copy of the document with all sections, see nature.com/documents/nr-reporting-summary-flat.pdf

Life sciences study design

All studies must disclose on these points even when the disclosure is negative.

Sample size	The study dataset comprised 57,416 British Bangladeshi and Pakistani individuals, aligned with the study objective of exploring cancer in this ethnic group. This cohort included 2782 participants with cancer across 33 different types, and 54364 non-cancer controls. Whole-exome sequencing data was available for approximately 76% of the cohort (n=43462). This represents the largest cancer cohort from this particular ethnic group in the UK, easily outnumbering similar cohorts in Genomics England 100K GP or UK Biobank.
Data exclusions	From the initial cohort of 57849 Genes & Health participants, 433 participants not from Bangladeshi or Pakistani ancestry-corrected ethnicity were excluded.
Replication	Using clinical data, age at cancer diagnosis distribution was replicated in the secondary cohort. The findings from exome-wide association study in the primary dataset underwent for cross-ancestry validation in a secondary dataset for 3 cancers with minimum number of participants in South Asian (Bangladeshi and Pakistani) group. Case numbers in secondary datasets were simply inadequate for meaningful validation of results at the level of other cancer types.
Randomization	There was no randomisation.
Blinding	There was no blinding.

Reporting for specific materials, systems and methods

We require information from authors about some types of materials, experimental systems and methods used in many studies. Here, indicate whether each material, system or method listed is relevant to your study. If you are not sure if a list item applies to your research, read the appropriate section before selecting a response.

Materials & experimental systems

n/a	Involved in the study	
☒	☐	Antibodies
☒	☐	Eukaryotic cell lines
☒	☐	Palaeontology and archaeology
☒	☐	Animals and other organisms
☐	☒	Clinical data
☒	☐	Dual use research of concern
☒	☐	Plants

Methods

n/a	Involved in the study	
☒	☐	ChIP-seq
☒	☐	Flow cytometry
☒	☐	MRI-based neuroimaging

Clinical data

Policy information about clinical studies
All manuscripts should comply with the ICMJE guidelines for publication of clinical research and a completed CONSORT checklist must be included with all submissions.

Clinical trial registration	This is not a clinical trial.
Study protocol	The data collection, processing, and analysis protocol is available in Methods section.
Data collection	Genes and Health December 2023 release.<br>Genomics England data release v19 (October 2024)
Outcomes	Primary outcome: Association between pre-existing medical conditions and incident cancers. Case-control study was conducted with logistic regression models developed for each cancer-morbidity pair.<br>Secondary outcome: Exploration of Demographic characteristics.

Plants

Seed stocks	Not applicable.
Novel plant genotypes	Not applicable.
Authentication	Not applicable.
