# Supplementary material for: Genetic and health determinants of cancer risk in Bangladeshi and Pakistani individuals in the UK
Source: Nat Commun. 2026 Jun 18;17:7910. doi: 10.1038/s41467-026-74415-z (PMC13443560; doi:10.1038/s41467-026-74415-z)
Supplement: Supplementary file 4 [file 41467_2026_74415_MOESM5_ESM.pdf]

nature portfolio

Peer Review File

Genetic and Health Determinants of Cancer Risk in Bangladeshi and Pakistani Individuals in the UK

Corresponding Author: Professor Claude Chelala

This file contains all reviewer reports in order by version, followed by all author rebuttals in order by version.

Version 0:

Reviewer comments:

Reviewer #2

(Remarks to the Author)
This study presents a well-executed and timely genome–phenome analysis of cancer risk in a large, deeply phenotyped British South Asian cohort, addressing a major gap in cancer genomics research for underrepresented populations. The integration of whole-exome sequencing with linked primary and secondary care records is a clear strength and enables more precise phenotyping than is typically possible in EHR-based studies.

The identification of ancestry-specific cancer–phenotype associations, rare coding variants, and gene-level signals—many of which are absent from existing genomic resources—provides important foundational knowledge for future research and clinical translation.
Methodologically, the analyses are sound and clearly described. While statistical power is limited for most cancer types and external validation is not currently feasible, these constraints are well acknowledged by the authors and reflect broader limitations in available non-European datasets rather than weaknesses in study design.

Overall, this manuscript makes a significant contribution to precision health and population genetics by demonstrating the value of substructure-aware analyses in genetically clustered populations. I support publication and believe this work will serve as an important reference for future studies in South Asian and other underrepresented populations.

(Remarks on code availability)

Reviewer #3

(Remarks to the Author)
In this paper, authors perform a pan-cancer exome-wide analysis to investigate genetic determinants of disease risk in the largely understudied populations of individuals of Bangladeshi and Pakastani ancestry living in the UK. Overall, the manuscript is well-written helps address the underrepresentation of these ancestral groups in cancer genetic epidemiology. It would benefit from clarification of the statistical points raised below.

Major Comments
1. In the comorbidity association analysis section, authors fit a logistic regression model for each cancer-phenotype pair. However, I have some concerns about the effect of the high rate of kinship here. Standard logistic regression assumes independent observations, but here we have high relatedness with many family units where individuals often share environments which can incur correlated comorbidities in addition to correlated cancer outcomes (from shared genetic risk). This could potentially lead to inflated type I error. Did the authors control for familial correlation or consider instead fitting a random effect models/logistic mixed model with kinship?
2. Exome data is quite limited in its ability to successfully capture granular ancestry components. Why did the authors elect to adjust for PCs derived from exome sequencing instead of those derived from the common variants in the genotyping array of 51,166 individual with array data? This would much more completely correct for population stratification in their cohort.
3. Did the authors consider calculating well-established polygenic scores for the cancers studied in individuals with available genotyping array data and then adjusting for this polygenic background in the rare variant analyses? Risk of

complex diseases like cancer can be modeled using a disease liability threshold framework in which both rare and common variants contribute to disease risk, and accounting for polygenic burden may improve interpretation. Specifically, recent work has demonstrated that polygenic background can modify the penetrance and phenotypic consequences of rare variants. I believe it would therefore be informative to evaluate whether rare variant effects vary across quantiles of the PGS distributions. There are also emerging methods (e.g., RICE) that jointly model rare and common variation and may further improve power and clinical interpretability of any significant findings.

Chapel et al., Polygenic backgrounds influence phenotypic consequences of variants in cells, individuals, and populations, Cell Genomics (2025)

Williams, J., Chen, T., Hua, X., Wong, W., Yu, K., Kraft, P., Li, X., and Zhang, H. (2024). Integrating Common and Rare Variants Improves Polygenic Risk Prediction Across Diverse Populations. Preprint at medRxiv.

Minor Comments

4. Line 126: I assume gAncestry-corrected ethnicity is just gAncestry + 6,312 self- reported ethnicities? Please similarly confirm what “adjusting for gAncestry data” is.
5. Line 147: Please confirm if these numbers are medians.
6. Line 216: Please define what topographically concordant refers to – I am not quite sure.
7. Line 269: Refers to single variants, but Supplemental Figure 2 does not appear to be relevant to this.
8. Line 377: What are these expansions on known cancer associations? Are they loci previously identified for each cancer or an alternative cancer?

(Remarks on code availability)
Very nicely documented code and analytical pipeline

Version 1:

Reviewer comments:

Reviewer #3

(Remarks to the Author)
I thank the authors for incorporating my prior comments and revising the manuscript accordingly. I believe these additions have strengthened the paper.

I have one remaining comment (which is more of a point of curiosity than a concern). In Supplementary Figure 6, within the gene-level analyses, there appear to be distinct bands of genes where the PGS-adjusted log-odds ratios are slightly negative while the original log-odds ratios are variable (approx −1 to ~8). Conversely, there also appears to be a band of genes where the original analyses show near-null effects, but the PGS-adjusted analyses display substantial variability in log-odds ratios. I am curious whether there are any shared characteristics among the genes that fall into these bands. I do not view this as a barrier to publication, but it may be interesting to comment on if the authors have any insight.

(Remarks on code availability)

Reviewer #4

(Remarks to the Author)
The manuscript presents a genome–phenome analysis of cancer risk in a large cohort of individuals of Bangladeshi and Pakistani ancestry in the UK, integrating whole-exome sequencing with linked electronic health records. The study addresses an important and timely gap in cancer genomics by focusing on underrepresented populations and leveraging deep phenotyping. The analytical framework combines comorbidity analyses, exome-wide association testing and gene-level burden approaches, complemented by polygenic risk score integration. Overall, the study is well-conceived, methodologically solid and of high relevance for precision medicine and population genomics. The authors have substantially improved the manuscript in response to reviewer comments, particularly with respect to modelling relatedness, addressing population structure and incorporating polygenic background.
Please clarify the following:
1. Despite limitations in power are acknowledged, the manuscript would benefit from a clearer quantitative discussion of expected detectable effect sizes across cancer types. This is particularly important given the relatively small number of cases for several phenotypes (as shown in Supplementary Figure 2)
2. Although gene-level and variant-level associations are reported, functional interpretation remains limited. The enrichment analysis (CGI/PharmGKB) is a useful step, but additional discussion on biological plausibility and potential mechanisms would strengthen the translational relevance.
3. The inclusion of PGS analyses is a strong addition. However, the manuscript could further elaborate on the clinical implications of limited interaction effects, whether findings suggest additive vs non-additive architectures and implications for risk stratification in these populations.
4. The study is population-specific (Bangladeshi/Pakistani UK cohort). Even though this is a strength, a more explicit

discussion of transferability (or lack thereof) to other ancestries would be valuable.
5. Please clarify whether case definitions across cancer types are harmonized in terms of incident vs prevalent cases.
6. Please provide clearer justification for phenotype grouping strategy.
7. Some figures (especially supplementary) are dense and would benefit from improved readability/annotation.
8. Ensure consistency in terminology (e.g., “low-frequency” vs “rare” variants).

(Remarks on code availability)

Open Access This Peer Review File is licensed under a Creative Commons Attribution 4.0 International License, which permits use, sharing, adaptation, distribution and reproduction in any medium or format, as long as you give appropriate credit to the original author(s) and the source, provide a link to the Creative Commons license, and indicate if changes were made.
In cases where reviewers are anonymous, credit should be given to 'Anonymous Referee' and the source.
The images or other third party material in this Peer Review File are included in the article’s Creative Commons license, unless indicated otherwise in a credit line to the material. If material is not included in the article’s Creative Commons license and your intended use is not permitted by statutory regulation or exceeds the permitted use, you will need to obtain permission directly from the copyright holder.
To view a copy of this license, visit https://creativecommons.org/licenses/by/4.0/

Response to Reviewers

We thank the reviewers for their careful evaluation of our manuscript and for the constructive comments that helped improve the clarity and robustness of our analyses. Below we provide a detailed, point-by-point response to each comment. All changes have been incorporated into the revised manuscript and are indicated in the tracked-changes version.

Reviewer #2 (Remarks to the Author): Expert in cancer genetics and genomics, statistical genetics, underrepresented populations, and South Asian ancestries

This study presents a well-executed and timely genome–phenome analysis of cancer risk in a large, deeply phenotyped British South Asian cohort, addressing a major gap in cancer genomics research for underrepresented populations. The integration of whole-exome sequencing with linked primary and secondary care records is a clear strength and enables more precise phenotyping than is typically possible in EHR-based studies.

The identification of ancestry-specific cancer–phenotype associations, rare coding variants, and gene-level signals—many of which are absent from existing genomic resources—provides important foundational knowledge for future research and clinical translation.

Methodologically, the analyses are sound and clearly described. While statistical power is limited for most cancer types and external validation is not currently feasible, these constraints are well acknowledged by the authors and reflect broader limitations in available non-European datasets rather than weaknesses in study design.

Overall, this manuscript makes a significant contribution to precision health and population genetics by demonstrating the value of substructure-aware analyses in genetically clustered populations. I support publication and believe this work will serve as an important reference for future studies in South Asian and other underrepresented populations.

Response

We thank the reviewer for the positive evaluation of our study and for recognising its contribution to improving representation of South Asian populations in cancer genomics research.

Reviewer #3 (Remarks to the Author): Expert in cancer genetics and genomics, statistical genetics, and underrepresented populations

In this paper, authors perform a pan-cancer exome-wide analysis to investigate genetic determinants of disease risk in the largely understudied populations of individuals of Bangladeshi and Pakastani ancestry living in the UK. Overall, the manuscript is well-written helps address the underrepresentation of these ancestral groups in cancer genetic epidemiology. It would benefit from clarification of the statistical points raised below.

We thank the reviewer for the thoughtful and constructive feedback, which helped us strengthen both the methodological framework and clarity of the manuscript. We address each comment below.

Major Comments

1. In the comorbidity association analysis section, authors fit a logistic regression model for each cancer-phenotype pair. However, I have some concerns about the effect of the high rate of kinship here. Standard logistic regression assumes independent observations, but here we have high relatedness with many family units where individuals often share environments which can incur correlated comorbidities in addition to correlated cancer outcomes (from shared genetic risk). This could potentially lead to inflated type I error. Did the authors control for familial correlation or consider instead fitting a random effect models/logistic mixed model with kinship?

Response

We thank the reviewer for raising this important point. Given the high degree of relatedness within the cohort, we agree that accounting for familial correlation is essential to avoid inflation of association statistics. In the revised manuscript, we have replaced the original fixed-effect logistic regression framework with a mixed-model approach implemented in **SAIGE**, which explicitly models sample relatedness using a sparse genetic relationship matrix (GRM).

The sparse GRM was constructed using kinship estimates derived from **KING** based on genome-wide genotyping array data. The mixed model incorporates the GRM as a random effect, thereby accounting for both familial clustering and cryptic relatedness within the cohort.

This revised modelling framework is now used for the primary comorbidity association analysis, and is described in the **Methods section** (Comorbidity association analyses). We also verified that the overall pattern of significant associations remained consistent, but with attenuated effect sizes, after implementing the relatedness-aware framework. The updated results are reflected in **Results section** (Comorbidities associated with cancer)**, Figure 3,** and **Supplementary Tables 4 and 5**.

2. Exome data is quite limited in its ability to successfully capture granular ancestry components. Why did the authors elect to adjust for PCs derived from exome sequencing instead of those derived from the common variants in the genotyping array of 51,166 individual with array data? This would much more completely correct for population stratification in their cohort.

Response

We thank the reviewer for this comment. We agree that array-derived principal components provide a more comprehensive representation of population structure. We used principal components derived from exome sequencing data because these were available for the full cohort included in the ExWAS analyses. However, we have also included global ancestry assignments derived from genotype array data as categorical covariates to account for broad population background within the

Bangladeshi and Pakistani study population. On top of that, principal components calculated from exome sequencing data were incorporated to adjust for fine-scale genetic structure, rare variant clustering, and technical variation within the sequenced subset used for association testing.

To evaluate the consistency of ancestry information captured by the two datasets, we performed a concordance analysis comparing the first three ancestry-informative principal components derived from exome sequencing and genotyping array data in the subset of individuals with both datasets available. We observed strong concordance between the two sets of principal components (PC1 r = 0.995, PC2 r = 0.954, PC3 r = 0.935), indicating that exome-derived PCs adequately capture the major ancestry structure in this cohort.

A description of this analysis and a new supplementary figure illustrating the concordance between array- and exome-derived PCs have been added to the manuscript (**Supplementary Figure 8**). We also clarify in the **Methods section** (Single-variant association analyses) why exome-derived PCs were used for the primary analyses.

3. Did the authors consider calculating well-established polygenic scores for the cancers studied in individuals with available genotyping array data and then adjusting for this polygenic background in the rare variant analyses? Risk of complex diseases like cancer can be modeled using a disease liability threshold framework in which both rare and common variants contribute to disease risk, and accounting for polygenic burden may improve interpretation. Specifically, recent work has demonstrated that polygenic background can modify the penetrance and phenotypic consequences of rare variants. I believe it would therefore be informative to evaluate whether rare variant effects vary across quantiles of the PGS distributions. There are also emerging methods (e.g., RICE) that jointly model rare and common variation and may further improve power and clinical interpretability of any significant findings.

Response

We thank the reviewer for this valuable suggestion. In response, we conducted an additional set of analyses incorporating cancer-specific polygenic scores to evaluate whether low-frequency/rare variant or variant-aggregated associations were influenced by the broader polygenic background.

Specifically:

1. **PGS covariate adjustment:** We repeated the primary ExWAS analyses using the same analytical framework implemented in **REGENIE**, incorporating cancer-specific polygenic scores (PGS) derived from models available in the **Polygenic Score Catalog** as additional covariates.
2. **Stratified analyses:** Participants were divided into tertiles based on standardized PGS values (low, mid, high polygenic risk), and association tests were repeated within each stratum to evaluate whether low-frequency/rare variant effects differed across the polygenic risk distribution.

3. **Interaction testing:** We additionally fitted models including low-frequency/rare variant (variant-aggregate) × PGS interaction terms to formally test for modification of rare variant effects by polygenic background.

These analyses showed that:

- Effect size estimates were largely consistent between PGS-adjusted and unadjusted models, with only modest attenuation.
- The majority of significant associations remained significant after adjustment.
- Stratified analyses showed no systematic trend in effect sizes across PGS tertiles (Spearman $\rho = -0.14$, $P = 0.10$).
- No statistically significant variant (variant-aggregate) × PGS interactions were detected.

These results suggest that the rare variant associations identified in our study are largely independent of the polygenic component captured by current PGS models.

The full methodology and results of these analyses have been added to the **Methods** (Polygenic Risk Adjustment and Stratified Analyses)**, Results** (Impact of Polygenic Background on Exome-wide Associations)**, and Discussion sections**, and are illustrated in **Supplementary Table 15** and **Supplementary Figures 6 and 7.**

Minor Comments

4. Line 126: I assume gAncestry-corrected ethnicity is just gAncestry + 6,312 self-reported ethnicities? Please similarly confirm what "adjusting for gAncestry data" is.

Response

Yes. By ***gAncestry-corrected ethnicity***, we refer to ethnic origin primarily determined by genetically inferred ancestry (gAncestry) where this information was unambiguous, and by self-reported ethnicity for 6,312 participants (11%) whose gAncestry assignment was ambiguous or unavailable. We also note that the manuscript previously referred to "**adjusting** for gAncestry data"; this has been revised to "**correcting** for gAncestry data" to more accurately reflect the approach used.

This clarification has now been added at the same location in the **Results section**.

5. Line 216: Please define what topographically concordant refers to – I am not quite sure.

Response

We confirm that these values represent **median ages at diagnosis**. This has now been clarified in the text.

6. Line 216: Please define what topographically concordant refers to.

Response

We have clarified this definition in the revised manuscript. "Topographically concordant" refers to pre-existing conditions affecting the **same anatomical or organ system as the corresponding cancer**. The revised text now explicitly defines this concept when first introduced in the **Results section** (Comorbidities associated with cancer).

7. Line 269: Refers to single variants, but Supplemental Figure 2 does not appear to be relevant to this

Response

We thank the reviewer for noting this inconsistency. The figure reference has been corrected **(Supplementary Figure 4)** to ensure that the appropriate supplementary figure is cited.

8. Line 377: What are these expansions on known cancer associations? Are they loci previously identified for each cancer or an alternative cancer?

Response

We have clarified this point in the revised manuscript. In this context, "extensions" refer to genes that had previously been reported in association with **specific cancer phenotypes**, where our analysis identified associations with **additional cancer phenotypes** beyond those previously described. The text has been revised to explicitly state that these findings extend known cancer associations to additional cancer types.

Reviewer #3 (Remarks on code availability):

Very nicely documented code and analytical pipeline

We thank the reviewer for the positive feedback.

Response to Reviewers

We thank the reviewers for their careful evaluation of our manuscript and for the constructive comments, which have helped us improve the clarity and robustness of our paper.

Below, we provide a detailed, point-by-point response to the comments raised by the reviewers. All changes have been incorporated into the revised manuscript and are shown in the tracked-changes version.

Reviewer #3 (Remarks to the Author)

I thank the authors for incorporating my prior comments and revising the manuscript accordingly. I believe these additions have strengthened the paper.

We thank the reviewer for the positive evaluation of the revised manuscript.

I have one remaining comment (which is more of a point of curiosity than a concern). In Supplementary Figure 6, within the gene-level analyses, there appear to be distinct bands of genes where the PGS-adjusted log-odds ratios are slightly negative while the original log-odds ratios are variable (approx −1 to ~8). Conversely, there also appears to be a band of genes where the original analyses show near-null effects, but the PGS-adjusted analyses display substantial variability in log-odds ratios. I am curious whether there are any shared characteristics among the genes that fall into these bands. I do not view this as a barrier to publication, but it may be interesting to comment on if the authors have any insight.

Response

We thank the reviewer for this insightful observation. We further investigated whether genes contributing to the observed banding patterns shared specific characteristics. Genes within these regions were significantly enriched for ultra-rare variants, with singleton and ultra-rare variants (MAF≤0.01%) accounting for 95% of band genes compared to 44% in the remainder of the dataset, increasing to 97% vs 40% when restricting to nominally significant associations ($P \leq 0.05$). Consistent with this, effect size estimates for genes within these bands exhibited higher uncertainty, with larger standard errors compared to the rest of the dataset (median SE: 2.58 vs 2.18), and substantially higher among nominally significant associations (4.3 vs 1.0). We additionally observed that these patterns were more prominent in sex-specific cancers (e.g., breast, prostate, and gynaecological cancers), which likely reflects reduced effective sample sizes and lower variant carrier counts in these analyses. A concise summary of these findings has been added to the **Results section** (Impact of Polygenic Background on Exome-wide Associations).

Together, these findings suggest that the observed banding patterns arise from increased variability in effect size estimation due to low cumulative minor allele counts and sensitivity of rare variant aggregation tests to covariate adjustment, rather than shared biological characteristics. Importantly, these effects do not impact the overall high concordance between models ($r = 0.96$) or the conclusions of the study.

Reviewer #4 (Remarks to the Author):

The manuscript presents a genome–phenome analysis of cancer risk in a large cohort of individuals of Bangladeshi and Pakistani ancestry in the UK, integrating whole-exome sequencing with linked electronic health records. The study addresses an important and timely gap in cancer genomics by focusing on underrepresented populations and leveraging deep phenotyping. The analytical framework combines comorbidity analyses, exome-wide association testing and gene-level burden approaches, complemented by polygenic risk score integration. Overall, the study is well-conceived, methodologically solid and of high relevance for precision medicine and population genomics. The authors have substantially improved the manuscript in response to reviewer comments, particularly with respect to modelling relatedness, addressing population structure and incorporating polygenic background.

We thank the reviewers for their thoughtful assessment and constructive feedback. We are pleased that the reviewers found our study "well-conceived, methodologically solid and of high relevance for precision medicine and population genomics." Below we provide detailed responses to each comment.

Please clarify the following:

1. Despite limitations in power are acknowledged, the manuscript would benefit from a clearer quantitative discussion of expected detectable effect sizes across cancer types. This is particularly important given the relatively small number of cases for several phenotypes (as shown in Supplementary Figure 2)

We thank the reviewer for this important suggestion. We have performed comprehensive power analyses for both the exome-wide association study and comorbidity analyses, estimating minimum detectable effect sizes across cancer phenotypes at varying exposure frequencies.

For the comorbidity analyses, aggregate cancer phenotypes are powered to detect OR ≥ 1.3 for common conditions (20% prevalence), whereas site-specific cancers require larger effects (OR > 2.5–3.5). The observed associations showed an inverse prevalence-effect relationship.

For the ExWAS, aggregate phenotypes with > 1,800 cases are powered to detect $\beta \geq 0.9$ (OR ≥ 2.5) at MAF = 1%, while site-specific cancers with < 200 cases require very large effects ($\beta > 2.8$, OR > 16) at MAF = 1%. Observed single-variant associations showed a strong inverse MAF-effect correlation ($\rho = -0.72$, $P < 10^{-8}$) consistent with purifying selection.

We note that for cancer phenotypes with larger case numbers (e.g., > 300), our study is powered to detect moderate-to-large effect sizes (OR > 2–3), whereas for rarer cancers, only very-large-effect associations are likely to be detected. These analyses contextualise the absence of associations for certain cancers, support the observed enrichment of high-effect rare variant signals, and reinforce biological plausibility.

We have added quantitative power estimates to the **Methods section** (Power and detectable effect sizes), and integrated results relevant to inverse frequency-effect relationships into the **Results section** (Comorbidities associated with cancer, Coding variant associations, Gene-level variant aggregation and associations). We also expanded the limitation point in the **Discussion section**, and provided detailed results in **Supplementary Table 18.**

2. Although gene-level and variant-level associations are reported, functional interpretation remains limited. The enrichment analysis (CGI/PharmGKB) is a useful step, but additional discussion on biological plausibility and potential mechanisms would strengthen the translational relevance.

We thank the reviewer for this important point. While our primary aim was to systematically identify genetic associations in an underrepresented population, we agree that further discussion of biological plausibility enhances translational relevance. In the revised manuscript, we have expanded on the interpretation of key findings (**Discussion section**) by:

- Emphasising the relevance of certain loci (e.g., *NF1, ZNF155, FKBP6*) in established or emerging cancer-related pathways.
- Highlighting known or plausible functional roles including tumour suppression, cell migration, stress-response signalling, and cell proliferation.
- Discussing how pleiotropic associations identified through PheWAS provide supporting functional context,

3. The inclusion of PGS analyses is a strong addition. However, the manuscript could further elaborate on the clinical implications of limited interaction effects, whether findings suggest additive vs non-additive architectures and implications for risk stratification in these populations.

We thank the reviewer for this suggestion and have amended the **Discussion section** accordingly. Specifically:

- Genetic architecture: The absence of significant rare variant x PGS interactions and lack of a monotonic trend across PGS strata support an additive model, in which rare coding variants and common variant polygenic burden contribute largely independently to cancer risk.
- Clinical implications: These findings suggest that integrating rare variant and polygenic risk information may improve risk stratification, while also indicating that polygenic background does not substantially modify the penetrance of rare variants (i.e., PGS should not be used to attenuate rare variant risk in clinical settings).
- Caveats: We highlight limitations including the reduced portability of European-derived PGS to South Asian populations and the possibility that modest interaction effects may remain undetected due to limited power.

4. The study is population-specific (Bangladeshi/Pakistani UK cohort). Even though this is a strength, a more explicit discussion of transferability (or lack thereof) to other ancestries would be valuable.

We agree and have added an explicit limitation in the **Discussion section** addressing transferability:

"Finally, the ancestry-specific nature of this cohort should be considered when interpreting these findings. Differences in allele frequencies, linkage disequilibrium patterns and gene–environment interactions may limit generalisability to other populations, including other South Asian subgroups. While associations involving well-established cancer genes are more likely to generalise, novel signals driven by population-specific rare variants will require independent validation. Findings may be most transferable to South Asian diaspora populations in comparable Western settings."

5. Please clarify whether case definitions across cancer types are harmonized in terms of incident vs prevalent cases.

We thank the reviewer for this clarification request. We have clarified in the **Methods section** (Comorbidity association analyses) that cancer cases were defined based on earliest cancer diagnoses recorded in linked electronic health records, regardless of whether this occurred before or after recruitment into the Genes & Health cohort, and therefore included both incident and prevalent cancer cases. We also highlight that the temporal relationship with pre-existing conditions was explicitly modelled: only conditions with onset on or prior to the index date were considered as exposures. For ExWAS analyses, germline genetic variants represent fixed exposures present from birth and are therefore not subject to temporal confounding.

6. Please provide clearer justification for phenotype grouping strategy.

We have expanded the **Methods section** (comorbidity association analyses) to define and clarify the rationale for phenotype grouping. Specifically, we explain that:

- Cancer phenotypes were analysed at primary site, organ system and aggregate-level groupings to balance clinical specificity with statistical power
- Grouping was necessary to enable detection of associations for less frequent cancers
- Highly correlated cancer phenotypes were handled by retaining the association with the strongest statistical support to reduce redundancy.

7. Some figures (especially supplementary) are dense and would benefit from improved readability/annotation.

We thank the reviewer for this suggestion. We have revised majority of the figures, particularly in the Supplementary materials, to improve readability. The specific improvements include:

- Label clarity by increasing font sizes and use of **bold** font in labels and texts for all main article and supplementary figures
- Added annotations for supplementary figures; Improved annotations for main article figures.
- Redraw all main article figures to fit in A4 portrait or landscape size
- Separate Figure 4 into two figures to improve readability.

8. Ensure consistency in terminology (e.g., “low-frequency” vs “rare” variants).

We have reviewed the manuscript to ensure consistent terminology and adopted the following nomenclature:

- common variants (MAF > 5%)
- low-frequency variants (1% < MAF ≤ 5%)
- rare variants (MAF ≤ 1%).

Our ExWAS analyses were restricted to rare variants (MAF ≤ 1%), which we further subdivide into rare (0.1% < MAF ≤ 1%) and ultra-rare (MAF ≤ 0.1%, including singletons) where relevant to interpretation. As a result of this adoption, we have:

- changed 9 instances of "rare/low-frequency" or “low-frequency” variants to "rare" variants where the analysis included variants up to MAF = 1%
- changed 3 instances of "rare" to "ultra-rare" where specifically referring to variants with MAF ≤ 0.1%

MAF thresholds for rare and ultra-rare variants are defined at first mention in the **Results section**.
